# Quantitative Analysis of Microbicide Concentrations in Fluids, Gels and Tissues Using Confocal Raman Spectroscopy

**DOI:** 10.1371/journal.pone.0085124

**Published:** 2013-12-30

**Authors:** Oranat Chuchuen, Marcus H. Henderson, Craig Sykes, Min Sung Kim, Angela D. M. Kashuba, David F. Katz

**Affiliations:** 1 Department of Biomedical Engineering, Duke University, Durham, North Carolina, United States of America; 2 University of North Carolina Eshelman School of Pharmacy and University of North Carolina Center for AIDS Research, University of North Carolina, Chapel Hill, North Carolina, United States of America; 3 Department of Infectious Diseases, University of North Carolina School of Medicine, Chapel Hill, North Carolina, United States of America; 4 Department of Obstetrics and Gynecology, Duke University, Durham, North Carolina, United States of America; Glasgow University, United Kingdom

## Abstract

Topical vaginal anti-HIV microbicides are an important focus in female-based strategies to prevent the sexual transmission of HIV. Understanding microbicide pharmacokinetics is essential to development, characterization and implementation of efficacious microbicide drug delivery formulations. Current methods to measure drug concentrations in tissue (e.g., LC-MS/MS, liquid chromatography coupled with tandem mass spectrometry) are highly sensitive, but destructive and complex. This project explored the use of confocal Raman spectroscopy to detect microbicide drugs and to measure their local concentrations in fluids, drug delivery gels, and tissues. We evaluated three candidate microbicide drugs: tenofovir, Dapivirine and IQP-0528. Measurements were performed in freshly excised porcine buccal tissue specimens, gel vehicles and fluids using two Horiba Raman microscopes, one of which is confocal. Characteristic spectral peak calibrations for each drug were obtained using serial dilutions in the three matrices. These specific Raman bands demonstrated strong linear concentration dependences in the matrices and were characterized with respect to their unique vibrational signatures. At least one specific Raman feature was identified for each drug as a marker band for detection in tissue. Sensitivity of detection was evaluated in the three matrices. A specific peak was also identified for tenofovir diphosphate, the anti-HIV bioactive product of tenofovir after phosphorylation in host cells. Z-scans of drug concentrations vs. depth in excised tissue specimens, incubated under layers of tenofovir solution in a Transwell assay, showed decreasing concentration with depth from the surface into the tissue. Time-dependent concentration profiles were obtained from tissue samples incubated in the Transwell assay, for times ranging 30 minutes - 6 hours. Calibrations and measurements from tissue permeation studies for tenofovir showed good correlation with gold standard LC-MS/MS data. These results demonstrate that confocal Raman spectroscopy holds promise as a tool for practical, minimally invasive, label-free measurement of microbicide drug concentrations in fluids, gels and tissues.

##  Introduction

Currently, there are more than 34 million people living with AIDS globally; in particular, women continue to be disproportionately affected. In 2011, women accounted for approximately 58% of individuals living with HIV/AIDS in Sub-Saharan Africa [1]. Biological vulnerability, gender-based violence, socio-economic inequalities, and inability to negotiate safe-sex practices are key factors contributing to a higher HIV prevalence in women. These issues stimulate a growing need for women’s preventive strategies. In the continued absence of an effective and approved anti-HIV vaccine, topically acting microbicides have become a focus in female-based strategies to prevent the transmission of HIV from infected male partners.

A successful microbicide formulation establishes drug concentration distributions at target sites sufficient to remain prophylactic against HIV over time of exposure. Current lead delivery vehicles are vaginal gels [2] and rings [3]. After failures of earlier gel products, vaginal application of a 1% Tenofovir gel was shown to reduce the risk of HIV sexual transmission by 39% [4] in one (CAPRISA 004) of two studies [5,6], and a third Phase 3 trial (FACTS 001) is currently underway with this same formulation [7]. Tenofovir exhibits anti-HIV activity by inhibiting HIV DNA transcription after phosphorylation to Tenofovir diphosphate in host cells [8]. Recently, Tenofovir, in combination with Emtricitabine in an oral product termed Truvada^®^ (Gilead Sciences, Foster City, CA) was found to be effective in reducing the risk of contracting HIV, and has gained approval by the FDA for this indication [9].

Knowledge of microbicide drug pharmacokinetics (PK) and its determinants is essential to understanding microbicide functionality and to microbicide product development. Such information can guide a rational design process in which the product and its dosage regimen are optimized to achieve target drug delivery, i.e. requisite drug concentrations in target compartments over time of HIV exposure. In PK analysis of vaginal microbicides, drug concentrations are typically measured in cervicovaginal fluids, cervical and vaginal tissues and blood plasma [10-15]. These biological matrices are homogenized or mixed prior to measurement by analytical techniques such as HPLC and LC-MS/MS [16,17]. Central to this is the fidelity of sampling and measuring drug concentrations. For vaginal and cervical mucosal tissues (where the majority of contemporary microbicidal molecules act) this is particularly challenging, for a number of reasons, including:

1. Human vaginal mucosal tissue is obtained by punch biopsies, and these are limited in number and not readily standardized with respect to thickness of the excised tissue.
2. This tissue is homogenized, so that only weight-averaged values of concentration are obtained. Thus, these weight averages of concentration contain uncontrolled variability due to the non-controlled thickness of the tissue specimens.
3. Gradients in drug concentrations down through the epithelial and stromal layers have the consequence that concentrations measured in biopsy tissue homogenates overestimate the volume average values for the stromal layer [18] where infection is believed to occur.
4. The analytical measurements of microbicide drugs require sophisticated instrumentation and methodology. For example, the contemporary technique to measure Tenofovir and Tenofovir-diphosphate (its bioactive form) concentrations in tissue involves LC-MS/MS (liquid chromatography separation coupled with tandem mass spectrometry detection) in a highly-sensitive but expensive and sophisticated methodology that is not widely available.

During development and evaluation of candidate microbicide formulations, *in vivo* PK studies are frequently complemented by *in vitro* measurements of drug transport into and through specimens of excised human and animal cervicovaginal tissues [10,13]. After collection, these specimens are stored under a number of conditions prior to their testing for drug permeation, e.g. via snap freezing or immediate immersion in fixative and culture media. Clearly, the properties of these harvested tissues are not identical to those of the intact material *in vivo*. For example, there is no active vasculature that can clear drug in the lamina propria. The fine structure of the tissue may be altered; e.g. as cells swell the intracellular clefts may be reduced in size, and this could alter drug diffusion through the tissue [19]. Such differences between harvested and *in situ* tissue notwithstanding, there has been longstanding scientific use of *in vitro* assays of tissue permeability to various molecules, e.g. endogenous physiological modulators and drugs [20-22]. In the microbicides field, such assays are used to compare different vehicles as well as drugs themselves [23-25]. Typically, the rate of drug transport is characterized in terms of the permeability parameter. This is equal to the product of the drug diffusion coefficient and its partition coefficient (at the vehicle-tissue interface) divided by the thickness of the tissue specimen. An experimental configuration such as a Franz Cell is typically used in measuring permeability. Here, a fluid or gel overlayer (called the “donor compartment”) is placed over the tissue specimen supported by a porous membrane. Drug is sampled from a stirred “receiver compartment” beneath the membrane. The drug permeability is obtained by monitoring its concentration in the receiver compartment and assuming that it is a linear function of time. Although permeability is a useful parameter in understanding drug transport from a delivery vehicle into and through a tissue specimen, it is not a fundamental material property of the transport process. Rather, it is a derived parameter that depends upon three basic parameters: the diffusion coefficient for drug transport through the tissue; the partition coefficient at the vehicle-tissue interface; and the thickness of the tissue specimen. Strictly speaking, the deduced permeability also depends upon the resistance to drug transport of the porous membrane that supports the tissue, but this is usually neglected. Published data on permeability of Tenofovir through excised human cervical vaginal tissue specimens have shown considerable inter- and intra-patient variability [10]. The reasons likely relate to natural biological variability in the tissues and also technical variability in the performance of the permeability assay.

Clearly, there is a need for improved methodology that can sample local drug concentrations, e.g. in relation to depth, in specimens of mucosal tissue. This would improve our understanding of drug concentrations in biopsy specimens taken in PK studies and their interpretation with respect to anticipated prophylactic concentrations. It would also improve our understanding of the mechanisms of the drug transport process overall, which could translate into improved product design. For example, information on drug concentration distributions in tissue, with respect to depth and time, could be used to deduce the diffusion coefficient within that tissue (and compartments within it, viz. the epithelium and stroma). This parameter is the salient measure of the rate of drug transport, and is a critical input to mechanistic models of such transport [18]. A practical, user-friendly, and minimally invasive method for performing such measurements would thus benefit our understanding of the performance of microbicide formulations and be valuable in other contexts of drug delivery, as well.

An attractive approach to performing local drug concentration measurements is the use of Raman spectroscopy. This is an analytical technique that can be used to optically probe and quantify specific molecular components in heterogeneous and highly scattering matrices, such as tissues, in real time [26]. The Raman technique requires minimal sample processing and has been shown to be of practical value for measuring target molecular concentrations in a variety of biological matrices as well as in drug delivery vehicles [27,28]. Theoretically, when monochromatic radiation interacts with a compound, light may be absorbed or scattered. The majority of the scattered light undergoes elastic Rayleigh scattering, such that the scattered photon has the same energy content and wavelength as the incident photon. A small fraction of light (around 1 in 10^7^ photons), however, is inelastically scattered at a different wavelength to the incident light in a process called Raman scattering. The rotational and vibrational states of a molecule reveal the pattern of shifted frequencies, called Raman shifts, which are characteristic of the nature of specific chemical bonds present in the molecule. This renders Raman spectroscopy highly chemically specific, such that it can provide structural and chemical fingerprint information unique to the molecule.

 Over the past decade, Raman spectroscopy has become widely investigated for biomedical applications since it can reveal the chemical fingerprints of different target molecules, cells and tissues [29,30]. It is increasingly employed to detect target molecules in skin and tissues *in vitro* and has been applied to evaluate the composition of human skin (e.g., cancer diagnosis) *in vivo*. It is particularly useful for real-time diagnosis of diseases that alter the subcellular components of the tissues. Applications of Raman spectroscopy for detection of cancers of the breast, lung, skin and bladder, as well as vulnerable plaque in atherosclerosis have been documented [29]. Moreover, confocal Raman spectroscopy (CRS) has been utilized to examine the penetration and delivery of drugs into human skin *in vitro* and *in vivo* [31,32], and to assess biomolecular compositions and hydration of human skin [33]. These findings demonstrate the promising capacity of CRS as a rapid and noninvasive approach to track drug permeation in real time across tissues. Recently, a combined CRS and time domain optical coherence tomography (TD-OCT) system has been developed to measure depth-resolved Raman signals from tissue samples imaged by the OCT [34]. This technique integrates the depth-sensitive biomolecular specificity of CRS with the morphological analysis capability of OCT to provide noninvasive characterization of biological tissues. An example was cited for measurements on a “goat mucous membrane having an epithelial layer on top of a relatively thicker stromal layer.” 

Although the Raman spectroscopic technique has been extensively used in various biomedical applications, its implementation in the microbicides field has yet to be fully explored; to date, there has been only a single published paper. Bell et al. used Raman spectroscopy to detect Dapivirine in silicone elastomer intravaginal rings (IVR), mapping the drug distribution in both the sheath and core of the devices [35]. This information was useful in understanding the loading and rate of drug release from the rings.

The present study aimed to investigate the feasibility of using confocal Raman spectroscopy to detect microbicide drugs and to measure their local (i.e. position dependent) concentration distributions within fluids, gel vehicles, and tissue specimens. The study was an initial, technical evaluation of the application of this promising methodology 3 candidate microbicide drugs were evaluated: Tenofovir, Dapivirine and IQP-0528. All of these are being formulated and evaluated in vaginal gels and rings [13,36-38]. As noted above, there have been a number of Phase 3 trials of a vaginal gel that contains 1% Tenofovir. There are now two Phase 3 trials of an intravaginal ring loaded with Dapivirine [39,40]. IQP-0528 is a candidate microbicide drug in earlier stages of development [13,41]. Our analyses demonstrated the capabilities of Raman spectroscopy to identify all three of these drugs in tissue specimens. In so doing, we employed procedures used previously by other investigators for preparations of the drugs in media and their applications to excised tissues. As noted above, our *in vitro* manipulations of tissue exposure to drugs in different media, as those of our colleagues in other *in vitro* studies, likely altered tissue microstructure to some extent and, consequently, drug diffusion therein. Consistent with these companion studies, we did implement an assay of tissue cell viability, the MTT assay. Our goal was, thus, to conform to the standard of practice in the microbicides field while investigating a new analytical tool for application within that field. Such application could be for analyses of drug distributions within tissues that had been dosed with drug *in vivo* prior to collection (viz. PK studies), and for *in vitro* applications of drug to harvested tissue. We emphasized Tenofovir, and its gel formulation that has and continues to be tested in clinical trials. More is known about this drug than other microbicide compounds under evaluation. Tenofovir is the drug that has advanced the furthest towards demonstration of topically acting anti-HIV efficacy and regulatory approval of its gel as a topical anti-HIV product. Thus, for technical demonstration of the many of the features of applying Raman spectroscopy to microbicides, we focused primarily upon this drug. In so doing, we analyzed the clinical Tenofovir gel currently in a third Phase 3 trial, and also the gel used in the trials as a control for the Tenofovir gel. Both the Tenofovir and placebo gels are based on hydroxyethylcellulose. However there are differences in their compositions and, thence, in properties such as pH values. As such, these are not physicochemically matched gels, the control gel simply being devoid of the active pharmaceutical ingredient. However, such differences do not confound the context of our results, which are focused on the feasibility of using confocal Raman spectroscopy for these types of drug concentration measurements.

Our study proceeded in several stages.

• We evaluated the Raman spectra of the three drugs in solution. Here, we also included evaluation of the spectrum of Tenofovir Disphophate, which is the bioactive form of Tenofovir that is created by phosphorylation after it enters cells (see below).
• We incubated specimens of porcine buccal tissue with these drug-laden solutions, after which we obtained the Raman spectra in the tissue and identified spectral peaks unique to the drugs that were distinct from those of the tissue. In so doing, we monitored the viability of the tissue specimens that had been incubated with Tenofovir gel over time. This provided initial proof of principle that Raman can be used to detect the drugs in tissue.
• We incubated the Tenofovir clinical gel with tissue specimens and again identified Raman spectral peaks in the tissue that can be used to detect this drug. Here, the Raman spectrum in tissue was different from that after incubation with solutions; that is, gel components (primarily glycerol) had entered the tissue and altered its Raman signature vs. that after incubation with Tenofovir-laden solution. As a gel control, we also incubated tissue specimens with a hydroxyethylcellulose gel, devoid of glycerol, that is used as a placebo in current microbicide gel trials.
• We evaluated the linearity of the drug-specific Raman spectral peaks in tissue vs. drug concentration. Serial dilutions of drugs in solution were performed. Drugs were also incubated to equilibrium after thorough mixing into homogenized tissue specimens. Linearity of the relationships of Raman signature peak heights vs. drug concentrations was assessed, and measurement sensitivities and limits of detection were obtained. In these experiments, we also measured Tenofovir concentrations in the tissue specimens by a gold-standard validated method of liquid chromatography coupled to mass spectrometry (LC-MS/MS). Drug concentration measurements by LC-MS/MS were compared to those obtained from Raman, as a means to validate the Raman spectral approach to drug concentration measurements in tissue.
• We then began analysis of the application of Raman to measure the varying local concentrations of Tenofovir as it permeates into and through whole tissue specimens. A Transwell configuration was created in which a drug-laden fluid layer was placed over a specimen of excised tissue. This enabled drug to permeate out of the fluid, and into and down through a tissue specimen. At specified times after the onset of fluid-tissue contact, depth-specific Raman spectra were obtained (“z-scans”) using a confocal Raman microscope. Thence, the relationship of Tenofovir concentration vs. horizontal position (depth) in the tissue was evaluated.

Collectively, these different experiments provided evidence that Raman spectroscopy, in particular as applied confocally, is a promising methodology for measuring microbicide drug concentration distributions in fluids, delivery gels and mucosal tissue specimens.

## Materials and Methods

### Ethics Statement

This study used freshly excised porcine buccal tissues. The tissues were obtained with permission from a local abattoir (Hatley Family Farm, Hurdle Mills, NC). 

### Porcine Buccal Tissue Preparation

In our study, specimens of freshly excised porcine buccal tissue were used for *in vitro* analysis of drug transport into and through tissue. This material was used here as an *in vitro* substitute for human vaginal mucosa, owing to its relative abundance and structural similarity to human buccal mucosa, which is in turn histologically comparable to the vaginal mucosa [42-44]. Studies have shown that porcine buccal mucosa is less permeable to test molecules than human vaginal mucosa [45,46], and thus use of excised porcine buccal tissue provides a conservative estimate of drug permeation through human vaginal mucosal tissue. Although quantitative results for drug transport in the porcine tissue may not equate to those in human tissue, the material is structurally relevant for evaluation of the suitability of the Raman approach for measurements of drug concentration profiles. A high level of optical similarity between Raman spectra of human tissue and porcine tissue has been demonstrated [47-49].

Fresh porcine buccal tissue was harvested from domestic medium-sized pigs’ heads obtained from a local abattoir (Hatley Family Farm, Hurdle Mills, NC) immediately after slaughter. The tissue was transferred to the laboratory in ice-cold isotonic, aerated (5% CO_2_ and 95% O_2_) Krebs-Henseleit buffer (pH 7.4; Sigma cat. # K3753: 1.2 mM MgSO_4_, 1.2 mM KH_2_PO_4_, 4.7 mM KCl, 118 mM NaCl ), supplemented with 2.5 mM CaCl_2_, 25 mM NaHCO_3_, 4 mM HEPES, and 12.2 mM glucose. The buffer composition was modified to be closer to previously published formulations of the storage buffer for porcine buccal tissue [50,51]. The concentration of potassium ion exceeds physiologic conditions, and this buffer formulation may cause cell swelling which would reduce the intra-cellular spaces and slow diffusion of drugs within that space. To prepare tissue specimens, within 1 h postmortem the underlying connective tissue and fat were removed with a scalpel blade and surgical scissors. The mucosa was sliced with a Thomas Stadie-Riggs tissue slicer (Thomas Scientific, Swedesboro, NJ) to obtain consistent tissue thicknesses. Unless otherwise noted, skin biopsy punch devices (Acuderm, Fort Lauderdale, Florida) were used to cut freshly excised tissue slices into circular specimens (7-mm diameter; 1-mm thickness). The thicknesses of the tissue slices were measured with a micrometer to achieve the target thickness and minimize variations among them. To prepare tissue homogenates, the tissue was processed with an Omni General Laboratory Homogenizer for 5-10 minutes at a speed of 25000 rpm. No extra fluid was added. 

### Evaluation of the Viability of the Porcine Tissue Specimens

The viability of tissue specimens used in our protocol was assessed by the reduction of the tetrazolium salt 1-(4,5-dimethylthiazol-2-yl)-3,5-diphenylformazan (MTT; Sigma) by viable cells into formazan (a dark purple water insoluble compound). The MTT assay is a well-established viability and cytotoxicity assay [52,53] that measures cellular metabolic activity in tissue [54]. It is commonly used in the microbicides field to evaluate tissue specimen viability [10,23,24]. MTT is reduced to a formazan dye by the succinate-tetrazolium reductase (TR) system of the mitochondrial respiratory chain, which is active only in living cells [52,55]. Thus, the quantity of the dye produced directly correlates to the number of metabolically active, viable cells. Results are typically presented as a ratio of the optical density of the formazan product at 570 nm to the dry weight of the tissue specimen (TR index). 

Freshly excised tissue specimens (7-mm diameter; 1-mm thickness; n=3) were incubated while fully submerged in 1% Tenofovir clinical gel and maintained at 5% CO_2_ and 37 °C for different amounts of time (2, 6, 9, and 24 h) in a Heracell incubator (Thermo Scientific, Asheville, NC). Each time point was carried out in triplicate. After incubation, each tissue specimen was removed from the gel, washed five times with PBS (Invitrogen, Grand Island, NY) and transferred to a 48-well plate (Corning Incorporated, Corning, NY) for the MTT viability assay. 1 mL of MTT solution (0.5 mg/ml in Krebs buffer) was added to each well and incubated with the tissue specimen for 3 h at 5% CO_2_ and 37 °C. After 3 h, the tissue specimen was removed from the MTT solution and rinsed five times with PBS. The formazan product was extracted from the tissue by submerging the tissue sample overnight in 4 ml isopropanol on a rotating platform. The optical density of formazan was measured at 570 nm with isopropanol as a blank (Shimadzu UV1800 spectrophotometer). The tissue samples were dried overnight in a lyophilizer and then weighed. The TR index was then computed. The first viability assay (control) was conducted on fresh untreated tissue specimens and began 1.6 h post mortem (quickest possible time). In addition, the viability assay was implemented on deactivated tissue specimens as a control; these were boiled in water for 2h to inactivate enzyme activity prior to the assay. 

### Spectral Raman Measurements

Measurements were performed on two Raman microscope systems: a Horiba LabRAM ARAMIS Raman Microscope and a Horiba Xplora confocal Raman microscope (Horiba Jobin Yvon). Initial studies were performed with the former. The latter was then used in a Transwell assay (see below) to measure Tenofovir concentrations vs. depth in excised tissue specimens. Two external excitation sources were used with the Horiba LabRAM ARAMIS: a 785 nm NIR diode laser (Pilot series, Sacher Lasertechnik, Marburg, Deutschland) and a 633 nm He-Ne laser (Melles Griot, Carlsbad, CA), delivering approximately 20 mW and 5 mW at the sample level, respectively. The Horiba Xplora microscope employed an integrated internal laser of 785 nm, delivering a power of approximately 25-30 mW. NIR excitation was used with tissue because tissue autofluorescence and absorption of light by tissue is low in the NIR domain, thereby increasing depth of photon penetration in tissue and minimizing the effect of tissue heating. The spectrograph has an air-cooled CCD detector (Synapse, Horiba Jobin Yvon) and gratings of 600, 1200, 1800, and 2400 gr./mm. The objective lenses used in the study were 50x long-working distance (Long Working Distance M Plan Semi-Apochromat, LMPLFL50x, Olympus) and 10x (M Plan Achromat, MPLN10x, Olympus), operating in air, with numerical apertures of 0.5 and 0.25, respectively. The focal spot size of sampling at the tissue surface was measured to be approximately 18 x 10 and 72 x 76 µm^2^ for the 50x long-working distance and 10x objectives, respectively. The analysis of bulk fluids was performed with the 10x lens. The 50x lens was used to obtain the confocal depth-scanning measurements. An automated XYZ sample stage was moved axially to obtain z-scans of spectra vs. depth into a specimen. The approximate depth of focus was derived from the Full Width at Half Maximum (FWHM) of the axial intensity response curve of the silicon band at 520 cm^-1^. Depth of focus is affected by the optical properties of the material surrounding the object of interest. We measured depth of focus of the 50x lens on a polished silicon wafer in air (non-scattering medium), and when the wafer was covered by 240 μm of scattering tissue phantom (1% Intralipid and 2% Agar) for three different confocal pinhole sizes (Table 1). Spectral acquisition was obtained using LabSpec software (Horiba Scientific, Edison, New Jersey). Spectra were averaged from two to five accumulations, each with an acquisition time ranging from 15 s to 5 m. The conditions of measurement were adjusted depending on the type of the matrix to obtain a good signal-to-noise ratio without damaging the tissue samples during the period of measurement. The laser beam was directed onto the surface of each tissue specimen with a quartz cover slip (Esco Optics Inc., Oak Ridge, New Jersey), which was used to flatten the sample surface and prevent tissue dehydration. 

**Table 1:** Depth of focus (μm) of the Horiba confocal Raman microscope when using a 50x objective lens measured on a polished silicon wafer, comparing the wafer in air versus the wafer covered by a scattering tissue phantom layer of 240 μm.

Confocal hole (μm)	Air (non-scattering)	240 μm (scattering) tissue phantom
100	16	17-22
300	17-18	22-28
500	21-23	29-34

### Data Processing

Recording and processing of spectral images were performed using LabSpec software (Horiba Scientific, Edison, New Jersey). Acquired Raman spectra were smoothed using Savitzky-Golay smoothing, and baseline-subtracted by line-segment baseline subtraction. The curve fitting procedure was based on a nonlinear least-squares method to a Gaussian distribution, accounting for random noise in the measured spectra and determining peak positions and intensities. These spectral data were then exported to MATLAB (MathWorks, Natick, MA) for further analyses. 

Statistical analysis was performed using statistical software Statview 5.0 (SAS Institute Inc., Cary, NC). P values less than 0.05 were considered indicative of statistical significance. 

### Spectral Raman Signatures of Microbicide Drugs

Three microbicide drugs were evaluated: Tenofovir, Dapivirine, and IQP-0528. Tenofovir powder (Lot No.: 1278-H-1), Tenofovir clinical gel (MTN-003, Sublot Code: 581), and HEC clinical placebo gel (MTN-003, Sublot Code: 990) were generously provided by the CONRAD Program (Arlington, VA). Compositions of these gels are shown in Table 2. Both gels were formulated at a pH of 4 to 5 to mimic the normal vaginal pH [56]. The Tenofovir gel was designed by CONRAD to have a small amount of acidic buffer. The HEC placebo gel, however, was designed with no buffer. Their intent was that the placebo would not perturb the normal vaginal pH, but also be non-reactive when a study subject had sex and alkaline seminal plasma was introduced [57]. Dapivirine powder (Lot No.: 60416PIL03) and Dapivirine clinical gel (Batch No.: 09M02) were kindly supplied by the International Partnership for Microbicides (Silver Spring, MD). IQP-0528 powder (Lot No.: SJV-090224) and IQP-0528 clinical gel (Lot No.: WL121108) were generously provided by Imquest Biosciences (Frederick, MD). Serial dilutions in solutions, gels, and homogenized tissues were performed to identify spectral peaks of the drugs. Serial dilutions of Tenofovir, ranging from 0.01 to 1 %w/w, were prepared in alkalinized water, gel, and porcine buccal tissue homogenates. The alkalinized water (50 mM NaOH) was made by alkalinizing the High Performance Liquid Chromatography (HPLC) grade water (EMD chemicals, Darmstadt, Germany) with 5M NaOH stock solution (Sigma Aldrich, St. Louis, MO). Serial dilutions of Dapivirine, ranging from 0.003 to 0.05 %w/w, were prepared in 30:70 (v/v) water/isopropyl alcohol (Sigma Aldrich, St. Louis, MO) mixtures, in gels, and porcine buccal tissue homogenates. Serial dilutions of IQP-0528, ranging from 0.01 to 0.5 % w/w, were prepared in HPLC grade ethanol (>99.8%, Sigma Aldrich, St. Louis, MO), gels, and porcine buccal tissue homogenates.

**Table 2:** Compositions of clinical 1% Tenofovir gel and HEC placebo gel.

**Gel**	**Ingredient**
1% Tenofovir	Tenofovir (1% w/w)
	Edetate disodium
	Citric acid
	Glycerin (20% w/w)
	Methylparaben
	Propylparaben
	Hydroxyethylcellulose
	Purified water
HEC Placebo	Hydroxyethylcellulose
	Sodium chloride
	Sorbic acid
	Sodium hydroxide
	Purified water

Additionally, a stock solution (84.5 mM) of Tenofovir diphosphate in water (pH 7.4) was generously provided by Gilead Sciences (Foster City, CA). The solution was diluted to a working concentration of 4.08 mM (0.18 %w/w). The Raman spectrum of Tenofovir diphosphate solution in water was obtained for comparison with the spectra of Tenofovir in water and with the water control.

Before taking measurements, 120 µl samples of drugs (in either solutions, gels, or porcine buccal tissue homogenates) were loaded into each well of a 384-well, quartz bottom plate (Matrical Bioscience, Spokane, WA). The sample surfaces were flattened to allow maximum beam exposure and avoid interferences generated from surface roughness scattering. 

### Raman Spectra in Tissue Specimens

Fresh porcine buccal tissue specimens (7-mm diameter, 1-mm thickness) were incubated in either 1% Tenofovir clinical gel or HEC placebo clinical gel for 6 h in a Heracell incubator maintained at 37°C and 5% CO_2_. The Raman spectra were recorded to demonstrate the spectral peaks unique to Tenofovir that were distinct from those of the gel and tissue. 

### Comparative Measurement of Tenofovir Concentration using Raman and LC-MS/MS

Fresh porcine buccal tissue specimens (4mm x 4mm x 3mm) were immersed in isotonic Ringer’s solutions (Hospira Inc., Lake Forest, IL) containing different concentrations of Tenofovir (0.01, 0.03, 0.1, 0.3, 1 % w/w) and allowed to equilibrate for 24 h at 4°C. After 24 h, the tissue specimens and the surrounding fluid were collected separately and stored at −80°C overnight for further analysis. The final concentrations of Tenofovir in the surrounding fluid and in tissue were measured by Raman and LC-MS/MS. In order to measure concentrations in fluid using the Raman spectroscopic technique, residual fragments of tissue in final fluid samples were removed through centrifugation (twice at 12000 rpm for 10 minutes) using the Eppendorf 5417 C microcentrifuge. The supernatants were then collected for analysis.

To obtain quantitative analysis using LC-MS/MS [58], tissue samples were placed in tubes with water (10:1 v:w) and homogenized using a bead beating method developed on a Precellys® system prior to centrifugation to obtain an extract. The resulting tissue extracts or incubation solution containing Tenofovir were further diluted with water and mixed with isotopically-labeled Tenofovir (Tenofovir-IS) as an internal standard. Analytes were retained on a Waters Xbridge C18 (2.1 X 20 mm, 5μm) analytical column and eluted under gradient conditions. Data were collected on an Agilent® 6410 triple quadrupole mass spectrometer, using Agilent LC-MS/MS MassHunter Chromatography Software, using electrospray ionization with positive ion m/z transitions of 288/176 (Tenofovir) and 293/181 (Tenofovir-IS). Peak area ratios of Tenofovir to Tenofovir-IS were used for quantitative analysis. 

### Transwell Assay

**Figure 1:**
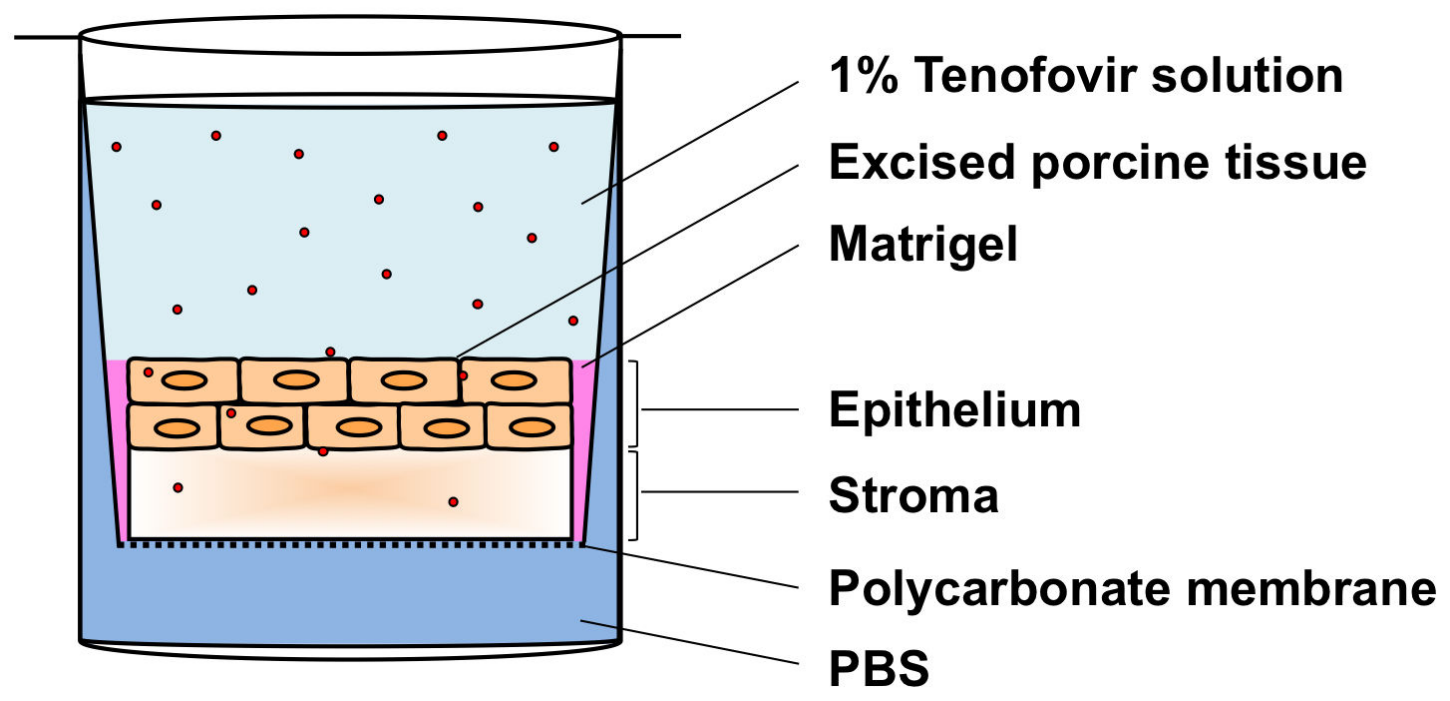
Schematic of Transwell assay for measuring Tenofovir transport into and through an excised tissue specimen. Circular porcine buccal tissue specimens were maintained in Transwell supports, under which was a bath of PBS to hydrate the tissue. Matrigel was used to create a gelatinous seal around tissue edges to prevent leakage. An isotonic solution of 1% Tenofovir in PBS was applied to the apical surface. The assay was maintained at 37°C and 5% CO_2_ in a Heracell incubator for 30 min to 6 h.

A Transwell assay was designed as a standard procedure to quantify time and depth-dependent drug concentration profiles in tissue (cf. Figure 1). The configuration had a reservoir of an isotonic solution of 1% Tenofovir in PBS on top of an excised porcine buccal tissue specimen (7-mm diameter, 1-mm thickness), under which was a bath of isotonic PBS to hydrate the tissue sample. 

For a given experiment, tissue samples were first fitted into a 24-well Transwell plate having an 8.0 µm pore-size polycarbonate membrane (Cat. No. 3422, Corning Incorporated, Corning, NY). Next, Matrigel (BD Biosciences, San Jose, CA) was applied around the edges of the tissue samples to minimize seepage along the samples’ sides. Timing of the experiment began when the Tenofovir solution was applied to the top of the tissue. PBS was underneath the polycarbonate membrane for the duration of the experiment. The Transwell setup was maintained at 37°C and 5% CO_2_ in a Heracell incubator for varying times, ranging from 30 min to 24 h. Once the desired incubation times were reached, the tissues and fluids were isolated and stored in a −80°C freezer overnight to stop the drug permeation process. Thawed tissue specimens were then subjected to confocal Raman scans at different depths from the surface down into the tissue proper (z-scans). Thawed fluids were also scanned. Quantification of Tenofovir concentrations was performed by interpolation, referencing to calibration curves of drug in tissue homogenates and fluids from the measurements described above.

## Results

### Spectral Raman Signatures of Microbicide Drugs

**Figure 2:**
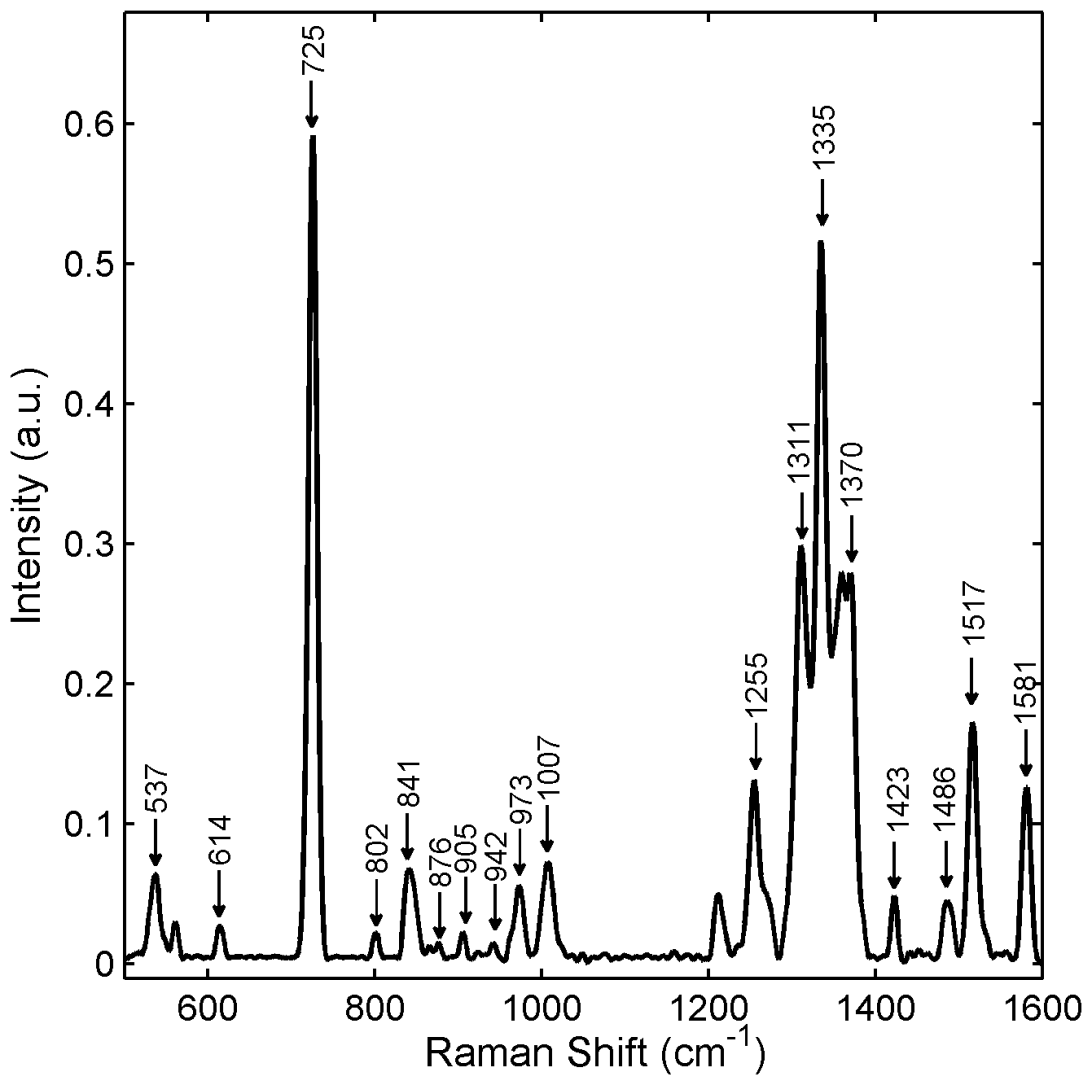
Raman spectrum of 1%w/w Tenofovir in water. 1% Tenofovir solution was prepared in alkalinized water (50 mM NaOH). The Raman spectrum of the Tenofovir solution was acquired using a Horiba LabRam ARAMIS Raman microscope with an excitation wavelength of 633 nm, 100μm slit width and a 10x objective lens. The acquisition time was 60s with 3 accumulations. Data were normalized to the area under the curve.

The first part of our study involved identifying characteristic Raman features unique to Tenofovir, IQP-0528, and Dapivirine. Figure 2 and Table 3 show spectral Raman signatures of Tenofovir along with vibrational assignments specific to these bands. Raman spectra of Tenofovir and Tenofovir diphosphate consist mainly of two sets of lines: ring vibrations of the adenine moiety and characteristic vibrations of the phosphate modes. Most of the strong vibrational features of Tenofovir are due to the adenine moiety with the strongest band located at 725 cm^-1^, while the phosphate group only contributes to weak vibrations. Tenofovir diphosphate, however, shows a prominent phosphodiester peak at 1112 cm^-1^ due to the symmetrical stretching vibrations of the PO_2_^ˉ^ combinations. Since this peak is absent in Tenofovir, it renders the spectra of Tenofovir and its diphosphate form distinguishable spectrally. 

**Table 3:** Identification of Raman peaks vs. vibrational modes for Tenofovir.

**Raman Shift ( cm^-1^ )**	**Tentative Assignment**	**References**
537 (w)**^*a*^**	δ(C-C=C)**^*b*^**	[74]
614 (vw)	δ(N-C-C)	[74]
725 (vs)	Adenine ring breathing (in-phase stretching)	[74,75]
802 (vw)	ν(C-C)	[74]
841 (w)	β(skel)	[74]
876 (vw)	γ,δ(N-H)	[74]
905 (vw)	r(NH_2_)	[74]
942 (vw)	δ(N-C=N)	[74]
973 (w)	Phosphate	[76]
1007 (w)	Phosphate	[76]
1255 (mw)	ν(C-NH_2_); ν(C=N); ν(C-N); δ(C-H)	[74,75]
1311 (ms)	ν(C-N); ν(C=N); δ(C-H)	[74-76]
1335 (s)	ν(C-N); ν(C=N); *w*(CH_2_)	[74,77]
1370 (ms)	δ(C-H); γ,δ(C-H)	[74]
1423 (w)	δ(N=CH); *w*(CH_3_)	[74,77]
1486 (w)	δ(NH_2_)	[74]
1517 (m)	δ(C-N-H); Imidazole ring	[74,75]
1581 (mw)	ε(NH_2_)	[77]

^a^ vs, very strong; s, strong; ms, medium-strong; m, medium; mw, medium-weak; w, weak; vw, very weak.

^b^ δ, bending; ν, stretching; r, rocking; w, wagging; ε, scissoring; β, in-plane; γ, out-of-plane; skel, skeleton vibration

In addition to those for Tenofovir, we found characteristic Raman bands for IQP-0528 and Dapivirine (Table 4). The strongest Raman features of IQP-0528, located at 1676 cm^-1^ and 549 cm^-1^, are due to the C=O stretching and in-plane bending modes of the uracil (thymine) moiety, respectively. Another strong Raman spectral feature of IQP-0528 was found at 1595 cm^-1^ as a result of the aromatic ring stretching vibration of the 1,3-dimethylbenzene ring moiety. Most of the strong vibrational features of Dapivirine are due to the benzonitrile moiety, while the mesitylene ring occurs as medium-to-weak Raman bands. 

**Table 4:** Salient Raman spectral peaks of microbicide drugs.

**Drugs**	**Raman peak** (**cm^-1^**) **^*a*^**	**Tentative assignment^*b*^**	**References**
Tenofovir	725 (vs)**^*c*^**	Adenine, in-phase stretching	[74,75]
	1112 (s)**^*d*^**	Phosphate, ν_s_(O$\dddot{-}$P$\dddot{-}$O)	[75,76]
	1311 (ms)	Adenine, ν(C-N), ν(C=N)	[74,75]
	1335 (s)	Adenine/ *w*(CH_2_)	[74,77]
	1370 (m)	Adenine, β,γ(C-H)	[74]
	1517 (m)	Imidazole ring, β(C-N-H)	[74,75]
IQP-0528	549 (vs)	Uracil,β(C=O)	[78,79]
	569 (m)	Uracil, β(C=O)	[78,79]
	670 (mw)	Uracil, γ(N-H)	[79]
	1009 (s)	Uracil, ν(ring)	[74]
	1299 (m)	Uracil, ν(ring)	[74]
	1595 (vs)	Aromatic ring, ν(C-C)	[80]
	1676 (s)	Uracil, ν(C=O)	[78]
Dapivirine	578 (m)	Mesitylene, β(arene)	[81]
	820 (mw)	Mesitylene, γ(C-H)	[81]
	980 (s)	Benzonitrile, ν(C-C)	[82]
	1174 (vs)	Benzonitrile, β(C-H)	[82]
	1610 (s)	Benzonitrile, ν(C-C)	[82]
	2223 (ms)	Benzonitrile, ν(C≡N)	[82]

^a^ vs, very strong; s, strong; ms, medium-strong; m, medium; mw, medium-weak; w, weak; vw, very weak.

^b^ β, in-plane bending; γ, out-of-plane bending; ν, stretching; *r*, rocking; *w*, wagging; s, symmetric; as, anti-symmetric

^c^ Underlined peaks are markers identified for microbicide detection in tissue.

^d^ Absent in Tenofovir

For a microbicide drug to be detectable in a practical, biologically relevant context, its Raman features must be sufficiently free from interference from solvent and tissue bands. Comparisons of the microbicide spectra and the tissue spectra enabled us to select the specific Raman bands that were strong and distinct from peaks due to the gel and tissue. These characteristic Raman features can be used as markers for detection of the microbicide drugs in tissue specimens that have been exposed to microbicide gels. They are located at 725 cm^-1^ for Tenofovir, at 1112 cm^-1^ for Tenofovir diphosphate, at 549 and 1595 cm^-1^ for IQP-0528, and at 1610 and 2223 cm^-1^ for Dapivirine. Details of all the Raman peaks and their putative relationships to vibrational modes are given in Table 4.

### Raman Spectra of Tenofovir in Tissue Specimens

**Figure 3:**
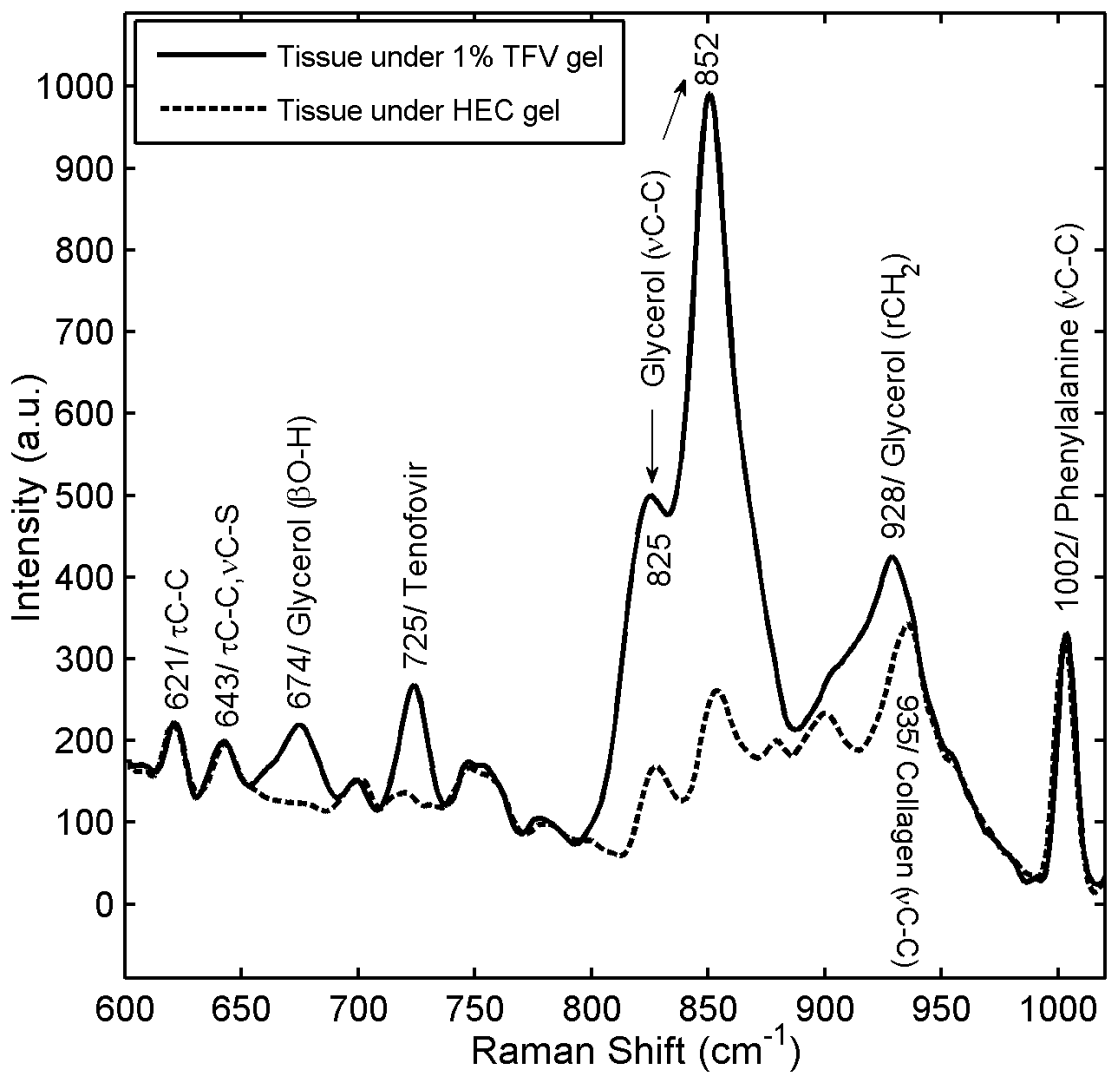
Raman spectra for clinical 1% Tenofovir (TFV) gel. Fresh porcine buccal tissue specimens (7-mm diameter, 1-mm thickness) were incubated by fully submerging them in 1% Tenofovir test gel (solid line) and HEC placebo gel (dotted line) for 6 h at 37°C and 5% CO_2_ in a Heracell incubator. The recorded Raman spectra demonstrated that the Tenofovir band was clearly distinguishable from Raman peaks due to the gel and tissue. β, bending; ν, stretching ; τ, twisting; r, rocking.

Figure 3 shows the Raman spectra of excised porcine buccal tissue specimens that were incubated while fully submerged in 1% Tenofovir gel or Placebo gel for 6 h. The most prominent spectral feature of Tenofovir, at 725 cm^-1^, was used as the marker to detect the drug in tissue. The signature peak of Tenofovir was clearly distinct from peaks due to glycerol in the test gel and tissue constituents. This Tenofovir gel contains glycerol (20%), as can be seen by strong Raman features at 825 and 852 cm^-1^ due to the C-C stretching vibration of glycerol. Although the Raman intensity of Tenofovir is weaker than the glycerol signal, the vibration of Tenofovir is present in the region where glycerol does not scatter strongly, enabling a clear distinction between the Tenofovir and the glycerol. 

### Spectral Concentration Dependence and Measurement Sensitivity

Successful Raman spectroscopic identification and quantification of a target analyte in a matrix derives from several interacting factors: (1) the analyte must exhibit spectroscopic peaks distinct from those of the matrix material; (2) the quantum efficiency of such peaks, which is driven by the excitation light intensity, must elicit a response that, when captured by the return optics, causes a signal which is sufficiently larger than the noise level of the detector; and (3) within the focal volume of the optical system, the Raman emission (peak height) must increase sufficiently in relation to analyte concentration. Tradeoffs amongst these factors are used to create several measures of the fidelity, or “sensitivity” of the measurements [59]. The signal-to-noise ratio (from factor (2)) establishes the limit of detection. The slope of the line of Raman peak height vs. concentration (factor (3)) gives the “calibration sensitivity.” These factors are all influenced by the optics of the system, scattering and absorption of light by the matrix, and possible reactions of the matrix with the analyte of interest. The “analytical sensitivity” is defined as the ratio of the calibration sensitivity to the standard deviation of the signals in a series of replicate experiments, and is a measure of the accuracy with which an unknown amount of chemical can be quantified. It can be improved by increasing the numbers of replicates, but is governed by the all three factors listed above.

**Figure 4:**
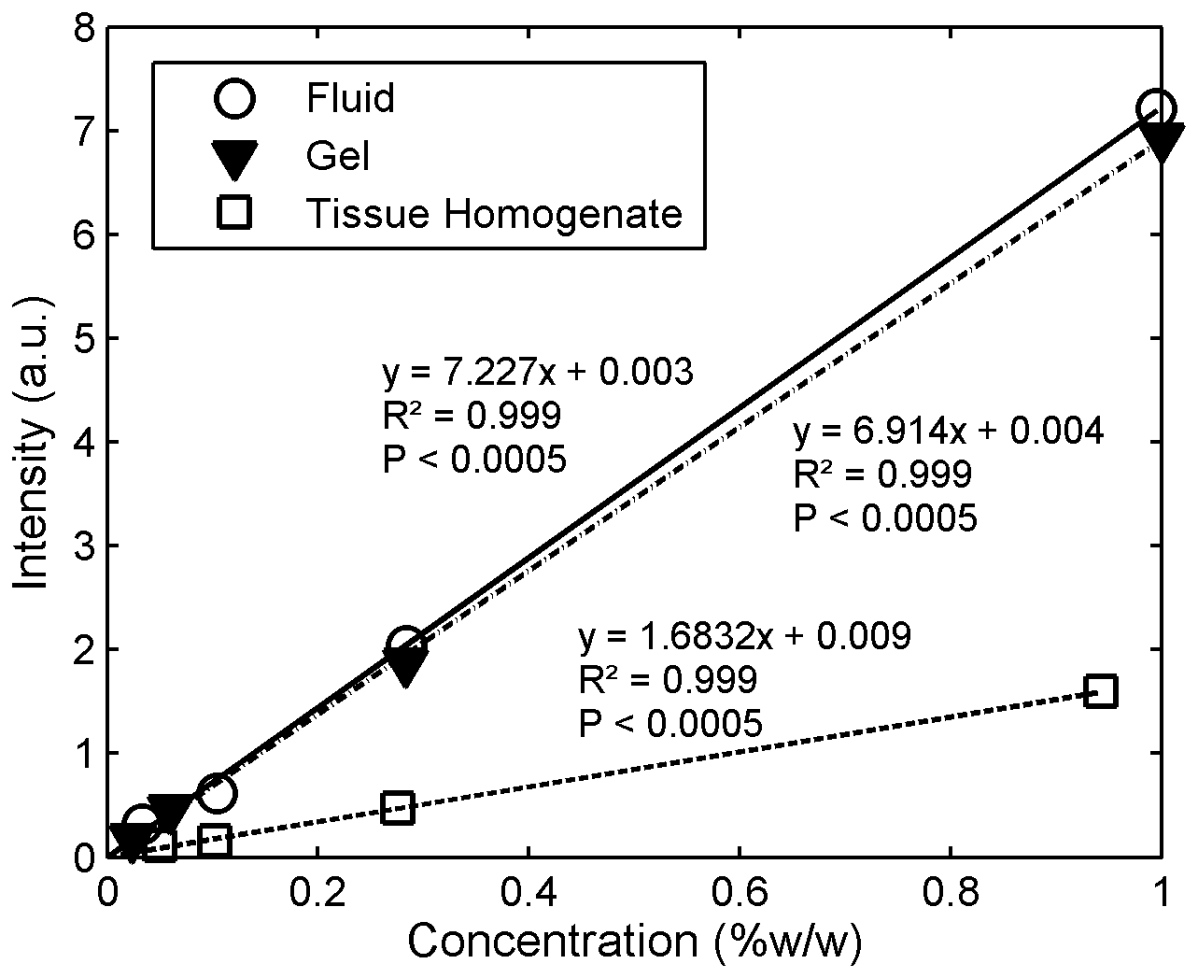
Relationships of signal intensity vs. **Tenofovir concentration in fluid, clinical gel, and tissue homogenate**. Serial dilutions of Tenofovir, ranging from 0.01 to 1%w/w, were prepared in alkalinized water (50mM NaOH), gel, and porcine buccal tissue homogenates. A strong linear dilution response (R^2^ ≥ 0.99, P <0.05) for concentrations of Tenofovir in solutions, gels, and tissue homogenates was observed. A saturation effect was not seen at the higher concentrations. This shows that Raman spectrometry is able to respond linearly to the physiologically relevant concentrations of drug. This linear dependence, in reverse, shows that Raman spectrometry can be used in practice to deduce the concentration of microbicide from the intensity of Raman peaks. Also, Tenofovir showed relatively higher signals in clear fluids and gels than in strongly scattering (opaque) tissues. The data shown represent the mean of 3-4 replicates.

**Figure 5:**
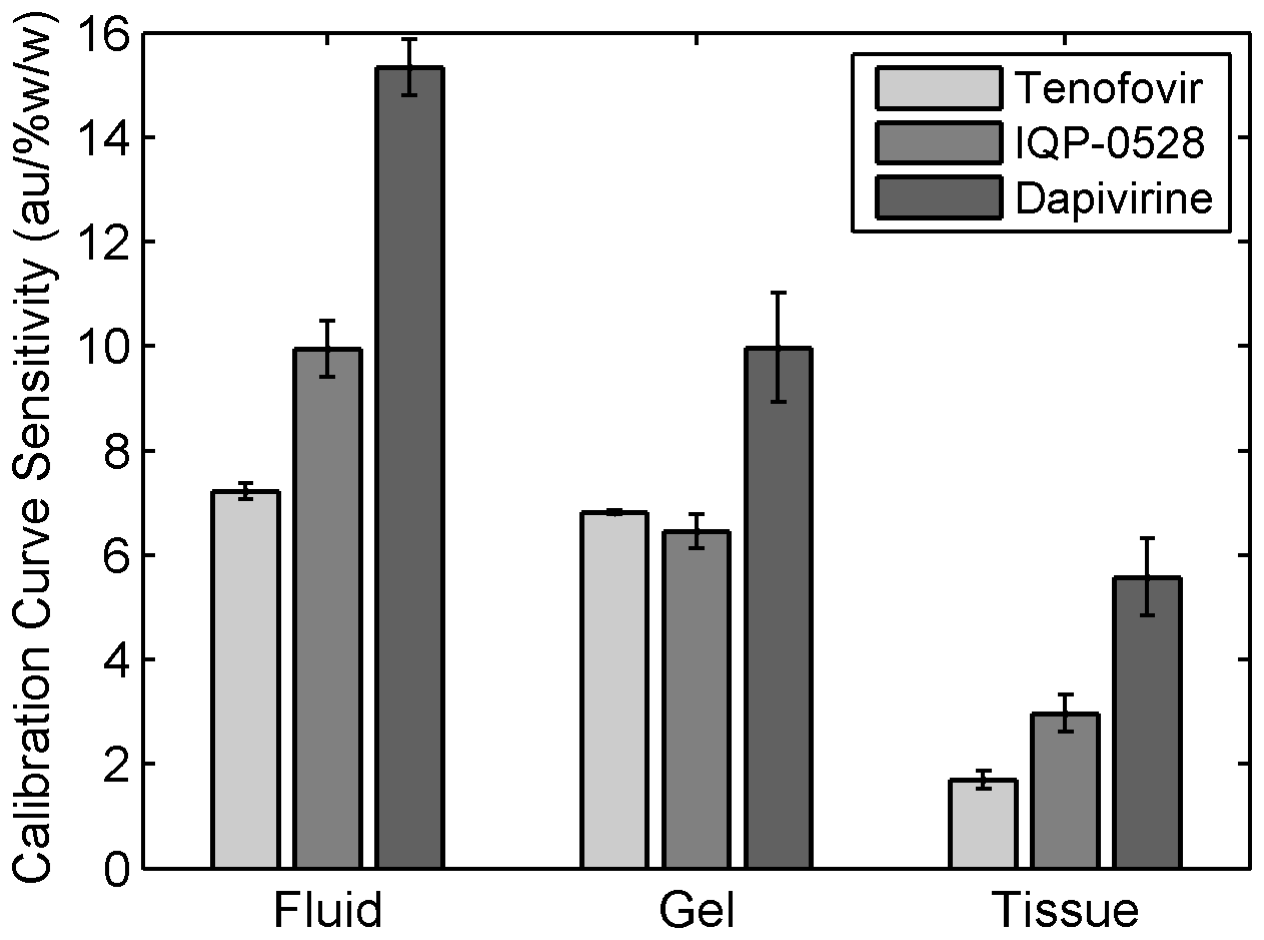
Calibration sensitivity for detection of Tenofovir, IQP-0528 and Dapivirine in fluid, clinical gel and tissue. The slopes, i.e. the incremental changes in intensity per change in concentration, of the linear dilution response curves represent the sensitivities of measurement. Response differences were observed for the three drugs in the different matrices. A significant difference in sensitivities among the drugs was observed (P<0.0001, two-way ANOVA with Fisher’s PLSD), and these relative sensitivities of drugs were dependent on the matrix in which they were measured (drug*matrix interaction, P <0.01). Fluids gave higher sensitivities than other matrices due to less background scattering. Dapivirine tended to have higher sensitivity than other microbicide drugs. The data shown represent the mean ± standard error of 3-4 replicates.

We found a strong linear dilution response (R^2^ ≥ 0.99) for concentrations of Tenofovir, Dapivirine and IQP-0528 in the three matrices—solutions, clinical gels, and tissue homogenates. The measured Raman intensity was linearly proportional to the concentrations of the active species. Figure 4 illustrates the linear response, showing the lines of Raman signal vs. concentration for Tenofovir in fluid, clinical gel and tissue homogenate. Figure 5 shows values of the calibration sensitivity for the 3 drugs in fluid, gel and tissue homogenate. Triplicate experiments were performed, in which standard errors were relatively low. However, we defer quantitative analysis of analytical sensitivity of this methodology to more extensive replicate testing in the future.

Differences were found in Raman signal for three drugs in different matrices. Using two-way ANOVA (drug and matrix as main effects) with Fisher’s PLSD post hoc analysis, statistical significances of these differences were examined. There was a significant difference in calibration sensitivity amongst the drugs (P<0.0001) and amongst the matrices (P<0.0001). In addition, the relative calibration sensitivities in detecting the drugs depended on the matrix within which they were measured (for the drug X matrix interaction, P <0.01). Generally, the calibration sensitivities were higher in fluids than in the other types of media due to less background scattering (because the fluids are transparent). In solution, the calibration sensitivity in measuring Dapivirine was significantly higher than that for IQP-0528 (P<0.0001), which had significantly greater calibration sensitivity than that for Tenofovir (P<0.01). In tissue, Dapivirine had significantly higher calibration sensitivity than IQP-0528 (P<0.05), which had numerically greater sensitivity than Tenofovir, although not quite at the level of statistical significance (P=0.057) at this sample size. In gel, Dapivirine had significantly higher calibration sensitivity than Tenofovir and IQP-0528 (P<0.05), for which the calibration sensitivities were not significantly different. Here, we note that the Tenofovir clinical gel is transparent; in contrast, the other two clinical gels are opaque due to light scattering, and this may have contributed to the differential sensitivities across the gels. 

**Figure 6:**
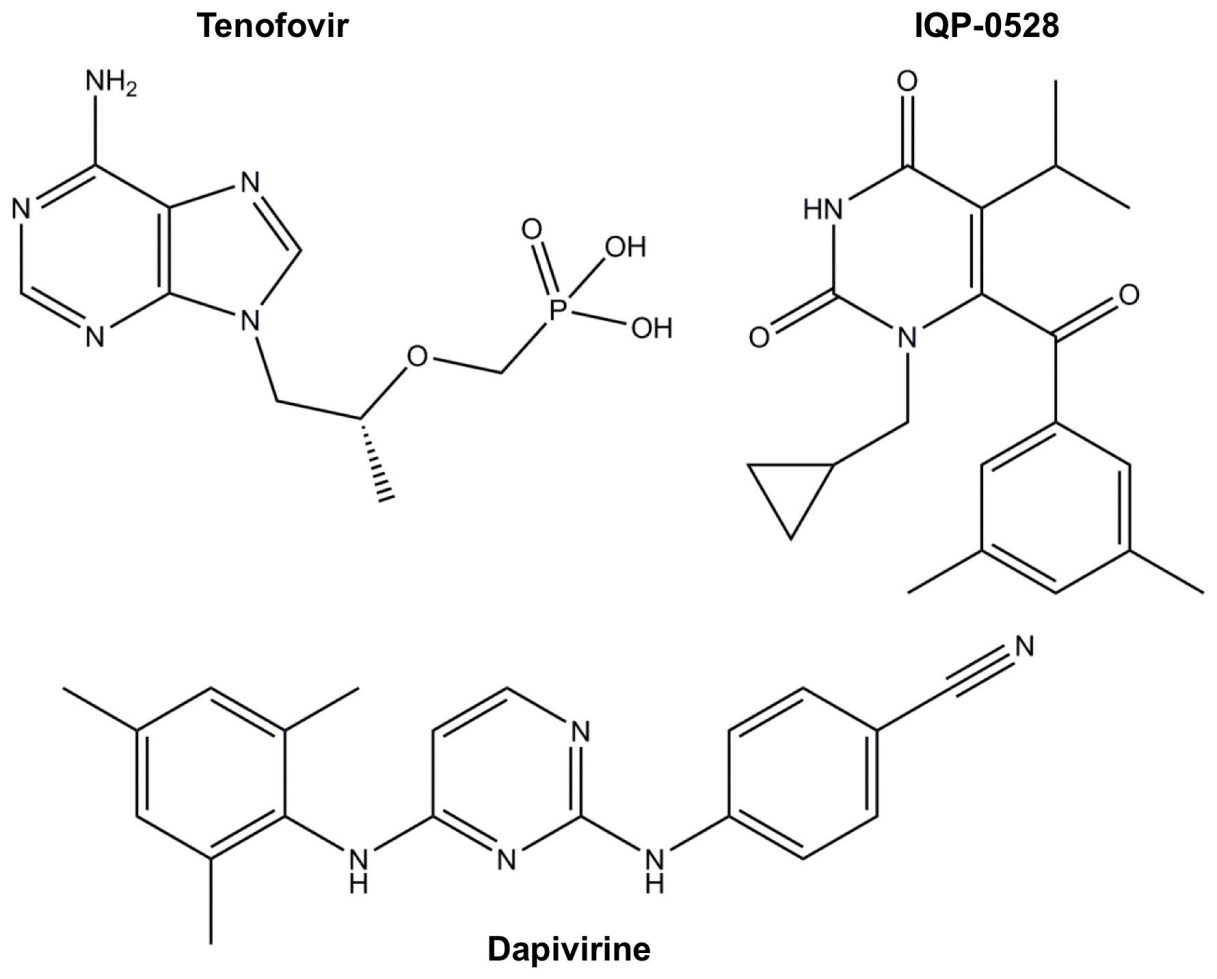
Chemical structures of Tenofovir, IQP-0528, and Dapivirine.

Overall, the calibration sensitivity in detecting Dapivirine was greater than those for the other two microbicide drugs. This can be explained by the effect of the Raman cross section of the molecules. Compared to the other two drugs, the chemical structure of Dapivirine (Figure 6) is less polar at rest. An incident photon causes a greater change in polarization and hence, gives relatively greater changes in Raman signal intensity per change in its concentration. Thus, Dapivirine has the highest sensitivity of detection. Tenofovir, however, is the most polar compound at rest; therefore, it has the weakest Raman intensity. 

To determine the lowest concentrations of the microbicide drugs that can be detected with acceptable reliability using the Horiba LabRam ARAMIS system, we performed an analysis of the limits of detection based upon the variability of values in blanks (i.e., zero concentration standards). The limits of detection were computed as mean blank value (n=7) plus three times the standard deviation (Table 5). These were found to be 0.01%w/w, 0.02% and 0.04% for Tenofovir detection in water, gel, and tissue, respectively. The limits of detection of IQP-0528 at 549 cm^-1^were found to be approximately 0.01% and 0.03% in ethanol and gel, respectively. The limit of detection of IQP-0528 at 1595 cm^-1^ was found to be around 0.04% in tissue. In addition, the limits of detection of Dapivirine at 2223 cm^-1^ were found to be approximately 0.003%, 0.006%, and 0.006% in isopropanol/water mixture, gel, and tissue, respectively. 

**Table 5:** Limit of detection (LOD) with the Horiba Raman system for Tenofovir, IQP-0528 and Dapivirine in different media.

**Drugs**	**Media**	**LOD (%w/w)**
Tenofovir	Water	0.01
	Gel	0.02
	Porcine tissue homogenate	0.04
IQP-0528	Ethanol	0.01
	Gel	0.03
	Porcine tissue homogenate	0.04
Dapivirine	70/30 iprOH/water	0.003
	Gel	0.006
	Porcine tissue homogenate	0.006

### Viability of Excised Tissue Specimens

**Figure 7:**
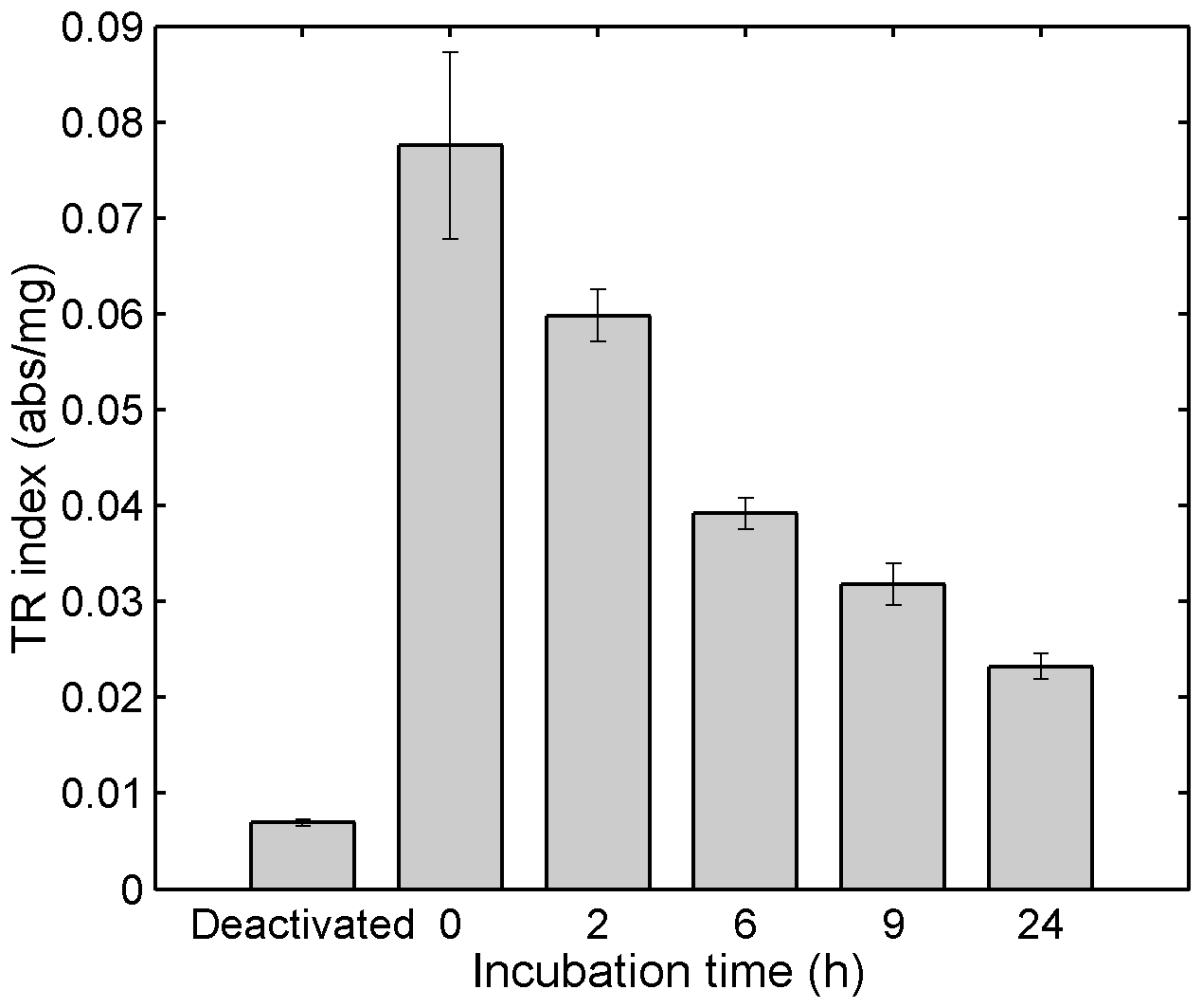
MTT assay of porcine tissue specimens fully submerged in 1% Tenofovir clinical gel at 37 °C. Freshly excised tissue specimens were incubated while fully submerged in 1% Tenofovir gel and maintained at 5% CO_2_ and 37 °C for different amounts of time. After incubation, the tissue specimens were removed from the gel, washed five times, and subjected to the MTT assay. Results were computed as a tetrazolium reductase index (TR index; absorbance per mg tissue) by dividing the absorbance the formazan product at 570 nm by the dry weight of the tissue specimen. Control samples were fresh untreated tissue specimens (1.6 h post mortem). To obtain deactivated (dead) samples, tissue specimens were boiled in water for 2h to inactivate enzyme activity. Data shown represent mean ±SE of 3-4 replicates.

The viability assay was conducted on tissue specimens that had been incubated while fully submerged in 1% Tenofovir clinical gel at 37°C over a period of 24 h. Mean TR indices of these tissue specimens were found to be 77%, 51%, 41%, and 30% of the control (fresh, untreated tissue) value after 2, 6, 9, and 24 h incubation with the gel, respectively (Figure 7). Although the TR indices of tissue specimens decreased over time over 24 h, the tissue viability at 24 h was relatively higher than that of the deactivated tissue, suggesting that some level of mitochondrial activity could still be maintained. A 50% decrease in TR index from control has been used as a limit for viability [50,51]. Using this cutoff value, tissue specimens incubated with 1% Tenofovir clinical gel were considered viable for up to 6 h when maintained at 37 °C.

### Measuring Tenofovir Concentrations vs. Depth in Tissue Using a Transwell Assay

**Figure 8:**
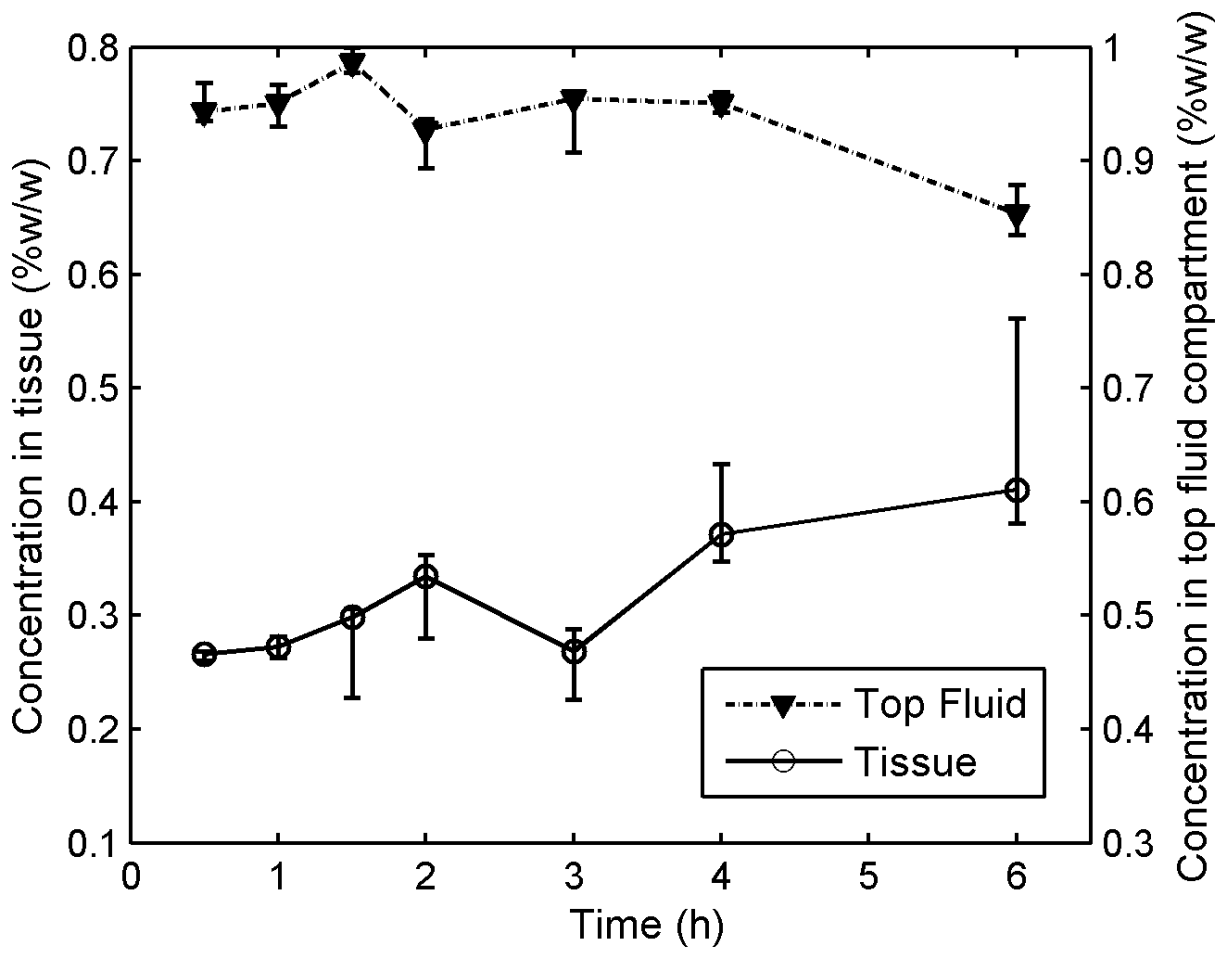
Tenofovir concentrations vs. **time in tissue and the top fluid compartment in the Transwell assay**. Transwell assay experiments were performed to characterize the transport of Tenofovir into and through tissue after microbicide application. The Transwell setup was maintained at 37°C and 5% CO_2_ in a Heracell incubator for different amounts of time, ranging from 30 min to 6 h. After incubation, the tissue (7-mm diameter, 1-mm thickness) and fluids were isolated and stored at −80°C. Thawed tissue specimens and fluids were scanned. Quantification of Tenofovir concentrations was performed by interpolation, referencing to calibration curves of Tenofovir in tissue homogenates and fluids (shown in Figure 4). Each data point gives the median, and the smallest and largest measurements in the 3 independent experiments (n=3).

**Figure 9:**
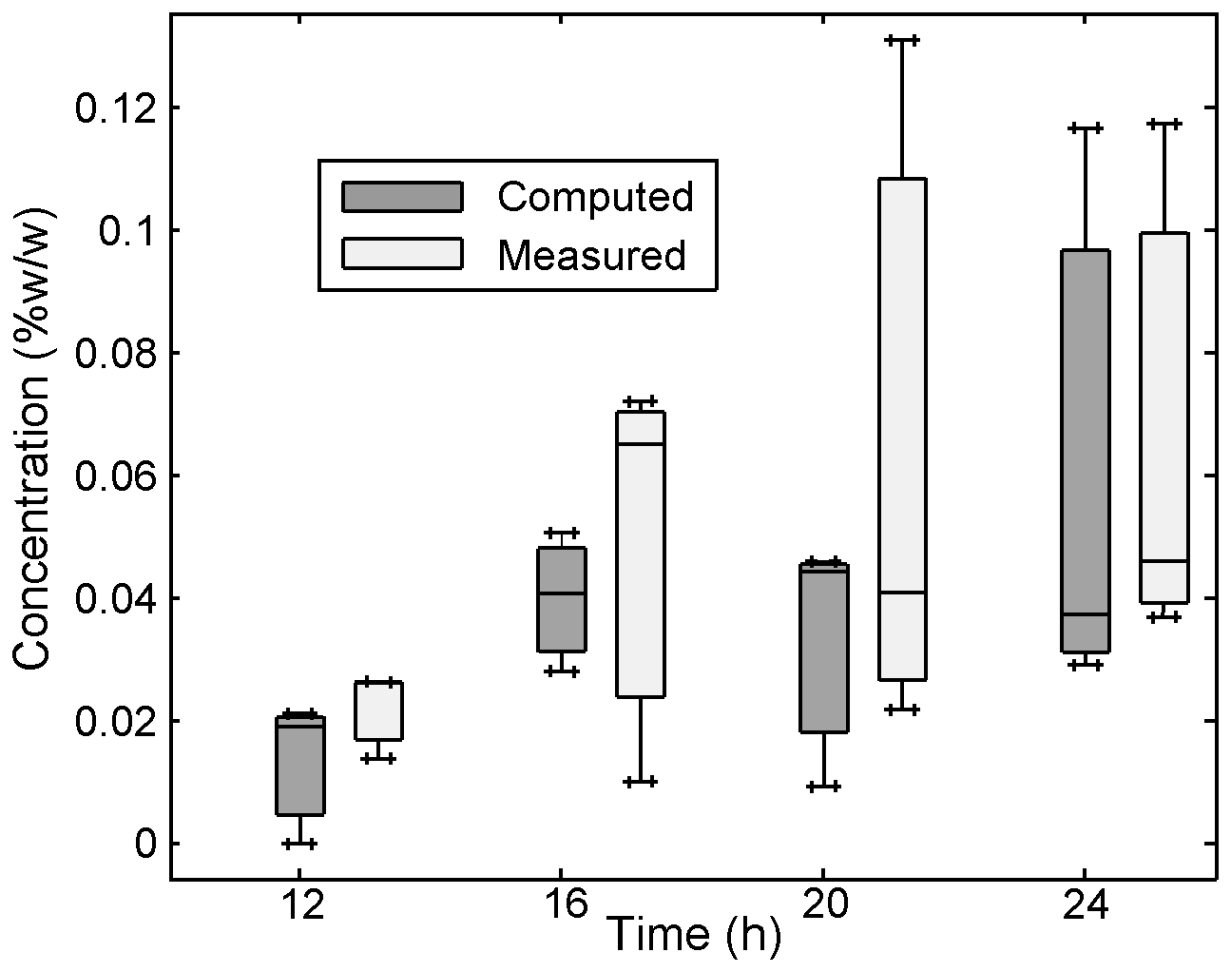
Tenofovir concentrations (measured vs. computed) in the bottom fluid compartment in the Transwell assay. A mass balance computation calculated the difference between the initial amount of Tenofovir in the fluid overlayer and the subsequent time-dependent sum of the total amounts of drug in the top layer and tissue. The good agreement between this mass balance prediction and the actual values measured supports the accuracy of the measurements. The box-and-whisker plot shows smallest value (lower bar), lower quartile (bottom of box), median (line through box), upper quartile (top of box), and largest observation (upper bar). The concentrations in the bottom fluid compartment from 0 to 9 h were not measurable (i.e., they were below the lower detection limit of the Raman quantification).

The *in vitro* Transwell assay was created as a standard method to evaluate time- and depth-dependent concentration distributions within tissue. Figure 8 shows Tenofovir concentrations vs. time measured in the top fluid compartment, and within excised porcine buccal tissue specimens that were incubated in the Transwell assay for varying times ranging from 30 m to 6 h. Tenofovir concentration in tissue increased with time and peaked at around 6 h. Figure 9 shows the result of a mass balance computation in which the sum of the amounts of Tenofovir in tissue and the top layer was subtracted from the initial amount of Tenofovir. Note the close agreement between results for this computation and the direct measurements.

**Figure 10:**
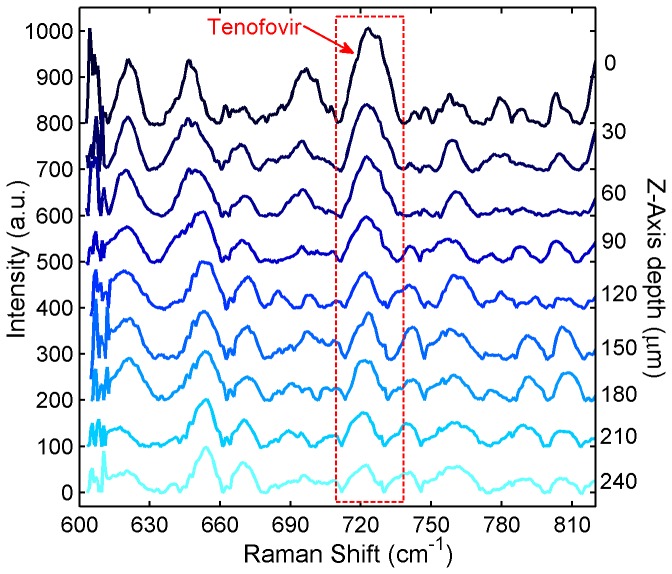
Z-scanning Raman spectra for Tenofovir penetrating excised tissue in the Transwell assay. 7 porcine buccal tissue specimens were incubated under an isotonic solution of 1% Tenofovir in PBS for 6 h in the Transwell assay. After incubation, the tissue was stored at −80°C overnight to stop the drug permeation process. Thawed tissue specimens were subjected to confocal Raman scans at different depths beneath the tissue surface to quantify Tenofovir distribution within the tissue specimens. The Raman spectra were acquired at different z-axis depths using a Horiba Xplora confocal Raman microscope with an excitation wavelength of 785 nm and an Olympus 50x long-working distance objective lens (N.A. =0.5). The acquisition time was 240 s for each spectrum. All spectra were normalized with respect to the tissue Raman band at 643 cm^-1^ and offset vertically for clarity of presentation.

**Figure 11:**
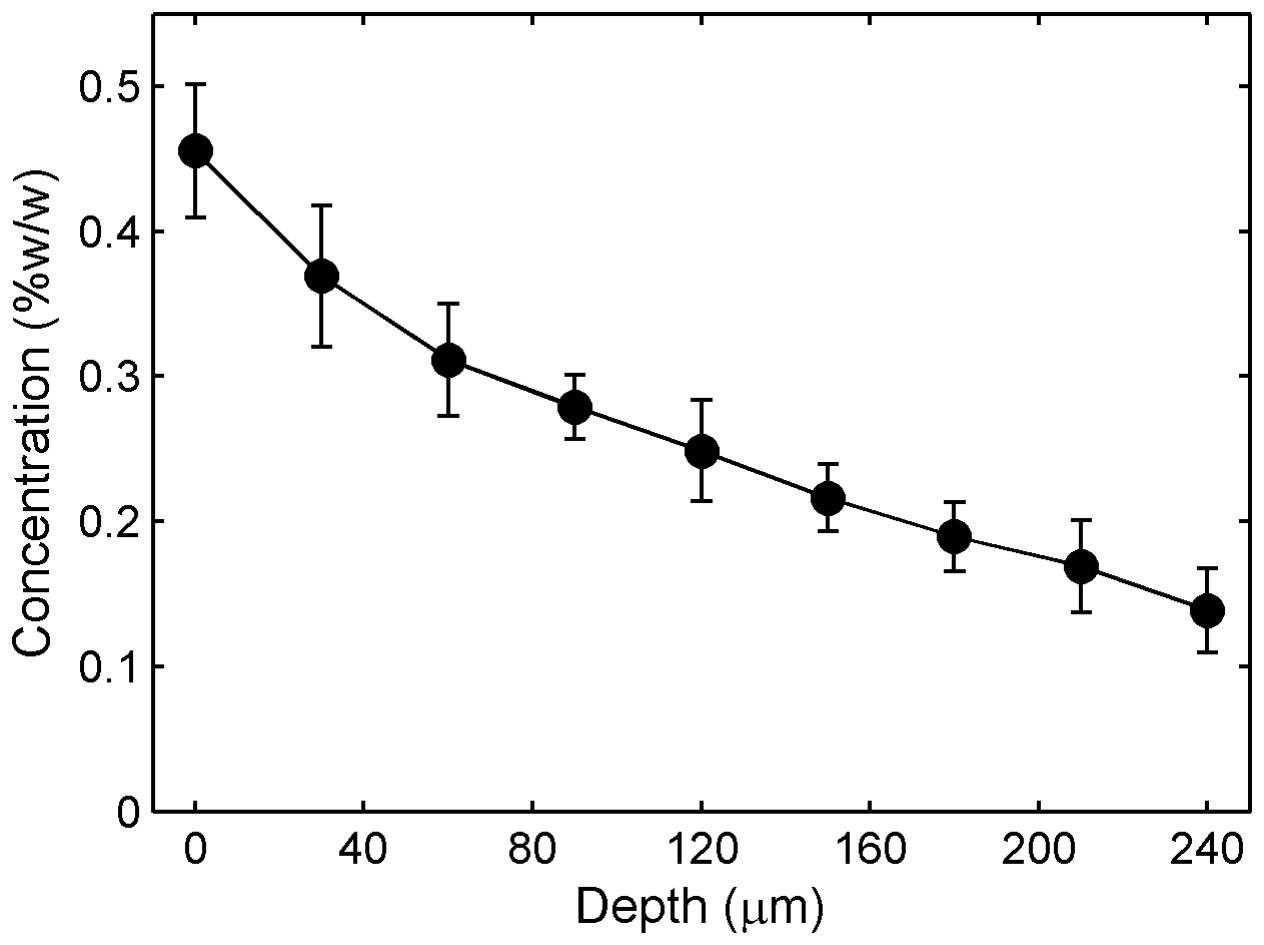
Tenofovir concentration vs. **depth in tissue in the** **Transwell assay**. Confocal Raman scans were performed on tissue specimens that had been incubated under a fluid layer (containing 1% Tenofovir) in the Transwell assay for 6 h (Figure 10). After baseline correction, the intensity of the Tenofovir Raman band was normalized with respect to the intensity of the tissue band at 643 cm^-1^. Normalized intensities were background-subtracted and fitted into a respective calibration curve of Tenofovir in tissue homogenate to deduce the concentration vs. depth profiles. The Raman measurements showed a decline in concentration as the depth into the tissue increased. Data shown give mean ± standard error of the mean (n=7).

Depth-scanning results of Tenofovir distribution from tissue specimens that were incubated in the Transwell assay for 6 h showed decreasing peak sizes from the surface down into the tissue (Figure 10). The intensity of the Tenofovir band at 725 cm^-1^ with respect to the intensity of the tissue peak at around 643 cm^-1^ (I_725_/I_643_) revealed the relative values of drug distribution at different depths. Moreover, quantitative analyses of Tenofovir concentrations showed a decline with depth into the tissue (Figure 11). These findings suggest that confocal Raman spectroscopy is able to distinguish different molecular features and differentiate varying concentrations among depths. 

Strictly speaking, “depth” reported here is the Z-axis translation of the automated XYZ microscope stage unit. When confocal Raman is applied to light-scattering matrices, this "depth" is not identical to the true location of the focal plane within the sample (see Discussion). 

### Comparisons of Tenofovir Concentration Measurements for Raman vs. LC-MS/MS

**Figure 12:**
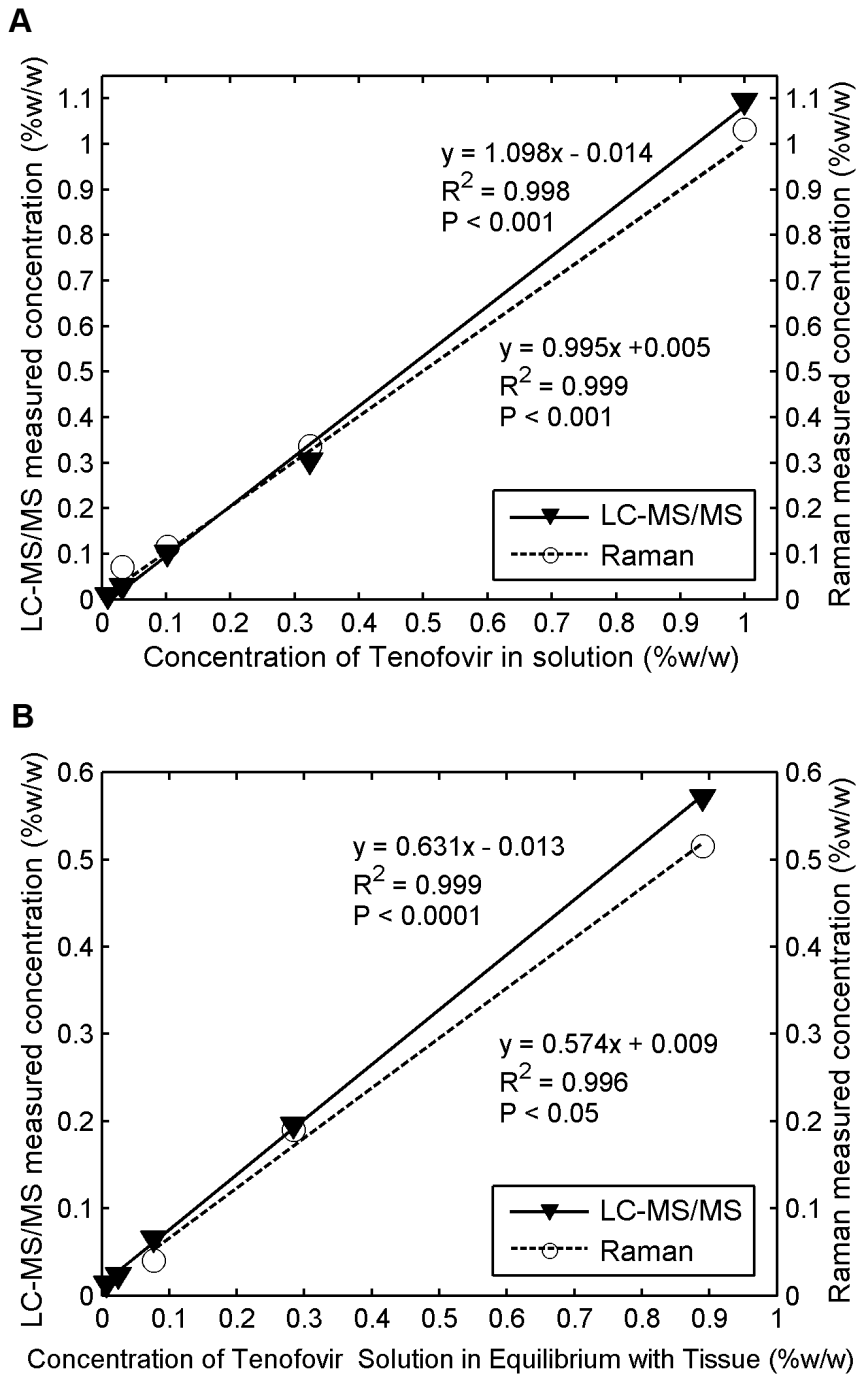
Comparative measurement of Tenofovir concentration using Raman vs. **LC-MS/MS in solution and tissue**. Freshly excised porcine buccal tissue specimens were incubated in serially diluted concentrations of Tenofovir in Ringer's solution and allowed to equilibrate for 24 h. After incubation, the tissue specimens and the surrounding fluid were collected and stored at −80°C overnight for analysis by both techniques: (a) The initial serially-diluted concentrations of Tenofovir in Ringer's solution measured by both techniques were plotted against its known concentrations in solution. These two techniques produced slopes that did not differ significantly (ANCOVA, P > 0.05); (b) the final concentrations of Tenofovir within tissue specimens measured by both techniques were plotted against concentrations of Tenofovir solution in equilibrium with tissue. ANCOVA also showed that the slopes obtained by both techniques were not significantly different (P > 0.05).

**Figure 13:**
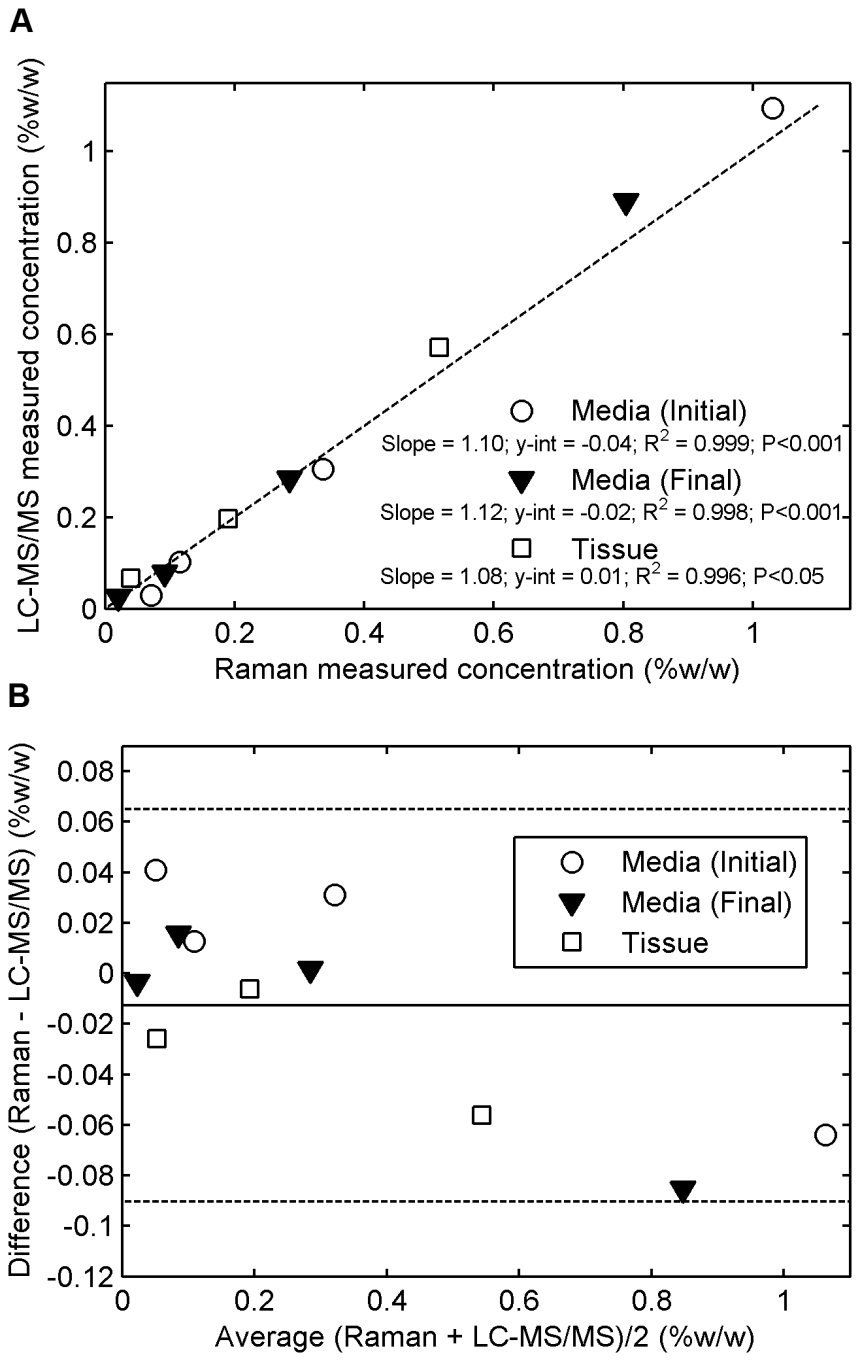
Agreement of Raman measurements of Tenofovir Concentration (Horiba instrument) with gold-standard **measurements by LC-MS/MS**. Serial dilutions of Tenofovir in Ringer's solution were equilibrated with freshly excised tissue specimens for 24 h at 4 °C. The Tenofovir concentration in fluid before (initial) and after (final) incubation, and the final concentration in tissue, were evaluated by both techniques: (a) the correlations between Tenofovir concentration values in fluid and tissue obtained from the Raman spectroscopic method vs. those derived from the validated LC-MS/MS technique. The dashed line indicates a straight 45-degree line (y=x) passing through the origin of the axes. (b) Bland-Altman analysis for determining the levels of agreement between the two measurement methods of Tenofovir concentrations in fluid and tissue. This gives absolute differences between the two measurement methods in relation to their average values. Solid lines represent mean differences, and dashed lines indicate the 95% confidence intervals.

We validated our Tenofovir measurements by comparison with ones obtained from the gold standard LC-MS/MS in fluid and tissue. Figure 12 shows good agreement between the two methods. They all produced linear regression lines with y-intercepts of zero, and slopes that did not differ significantly (ANCOVA, P > 0.05). Figure 13a demonstrates a good linear correlation between the Tenofovir concentrations obtained from the two methods. The y-intercepts were not statistically significantly different from zero (ANOVA, P > 0.05), and the slopes were not significantly different from one at the 5% significance level. Figure 13b presents comparisons illustrated via the Bland-Altman approach for determining the levels of agreement for concentrations obtained from the two methods [60]. These results show that across the range of Tenofovir concentration studied (0.01 to 1.00% w/w), the concentrations determined by Raman spectroscopy fall within ± 0.08% w/w (two standard deviations) of the measurements obtained from the gold standard LC-MS/MS technique.

## Discussion

We explored the feasibility of applying confocal Raman spectroscopy as a useful analytical technique that can contribute to pharmacokinetic and drug transport analyses. Our focus was on applications to vaginal delivery of topical anti-HIV drugs. The Raman technique was applied to characterize three current microbicide drugs and to measure their concentrations in fluids, clinical gels, and excised tissue specimens. Here, we utilized methods of tissue and drug preparation common in the microbicides field, and in others where drug permeability in tissue is evaluated, as well. Tissue fine structure in such specimens *in vitro* may not be identical to that of intact tissue *in vivo*, and this could alter drug transit rates. In this regard, the confocal Raman spectroscopic technique could be used in quantitative evaluation of how details of tissue harvesting and preparation affect such drug transit rates. Indeed the direct depth-dependent measurements with the Raman based technique could well be more precise than traditional approaches that rely upon physical sectioning of tissue specimens. 

Due to its highly chemically-specific nature, Raman spectroscopy was able to distinguish all the microbicide drugs from an optically scattering and absorbing tissue, which is itself a heterogeneous mixture of molecules and supramolecular structures. In fact, a high level of optical similarity between Raman spectral backgrounds of human tissue and porcine tissue has been observed [47-49], suggesting a similar biochemical composition of the two tissues. We also observed a strong linear dependence of the Raman peak intensities over ranges of microbicide concentrations in the three types of media. The linear dependence of Raman peak intensity on the number of molecules involved in the scattering process per unit volume is well established [61]. This renders Raman spectroscopy a useful technique for gaining both qualitative and quantitative information about the molecular and structural composition of a sample. Further, it motivates the use of Raman for quantitative measurements of concentrations of molecules.

Our emphasis was on Tenofovir. This is the microbicide drug that has received the greatest study to date and, pending results of the current FACTS 001 clinical trial, could be the first anti-HIV microbicide approved for vaginal application. We found a spectroscopic distinction between Tenofovir and Tenofovir diphosphate, the bioactive form of Tenofovir, which is phosphorylated from Tenofovir after it enters CD4+cells or macrophages. It is likely, however, that the actual Tenofovir diphosphate levels in mucosal tissue after *in vivo* Tenofovir administration would be too low to be detectable using Raman spectroscopy. For example, it was found that Tenofovir diphosphate concentrations in vaginal biopsies were only about 5-8% of the Tenofovir concentrations [36,58,62]. However, the level of local Tenofovir diphosphate can be objectively estimated from the local Tenofovir concentration, since there appears to be a linear correlation between the levels of the two forms of Tenofovir present in tissue [63]. This relationship could help in estimating the local Tenofovir diphosphate concentration in tissue from the spectroscopically measured Tenofovir concentration using Raman spectroscopy.

We validated our Tenofovir measurements with gold standard ones obtained using an LC-MS/MS method validated according to FDA criteria [16,64] in fluid and tissue samples. Good linear relationships between two methods over a range of Tenofovir concentrations were demonstrated. Measured concentration values were tightly scattered about statistically significant linear regression lines that passed through the origin.

We implemented a standardized Transwell assay as an *in vitro* model system to quantify drug permeation into and through tissue specimens. In our initial application here, the assay was constructed with a drug-loaded liquid layer over the tissue. However, this configuration can be extended to apply a gel layer of defined thickness over the tissue; our group has experience in creating such layers in Transwell assays involving HIV migration rates [65]. The approach to drug transport here is more informative than traditional permeability assays [66] in that it quantifies concentration in the tissue as a function of time during the course of an experiment. The Raman-transwell approach also shows promise as a means of measuring the local concentrations of Tenofovir at different depths as it permeates into and through tissue specimens. In fact, our z-scans in the Transwell experiments detected Tenofovir to a depth of 200+ µm (see next paragraph). Such information on drug concentration distributions could then be processed to compute values of drug diffusion and partition coefficients, which are fundamental transport parameters that govern the process of drug release from a gel and transport into and through the tissue [13,67]. These parameters can be used in predictive computations, using compartmental models, of microbicide pharmacokinetics, that can aid the understanding of the mechanisms of microbicide drug delivery and, thus, be used in product design and evaluation [18]. 

As noted earlier, a major challenge in confocal Raman spectroscopy, especially when working with complex biological tissues, is to accurately determine the depth resolution and the penetration depth [68-72]. Highly scattering or turbid materials such as biological tissue have spatially inhomogeneous refractive indices that contribute to local uncertainties in depth profiling. A conventional microscope illuminates the focus with a cone of light whose rays travel through many paths through the matrix to reach the focal point. This averages inhomogeneities in the matrix to some extent. In contrast, the type of confocal microscope used here has a single laser beam for illumination that may be only 100 microns in diameter, smaller than the sizes of some of the inhomogeneities. This beam has a single path through the matrix which can deviate significantly from a straight path, and it does not have the benefit of averaging. 

This topic has been explored in the literature. For example when testing confocal Raman imaging, using multilayered polymeric film systems with refractive indices similar to those of human skin, Xiao et al found that the penetration depth was underestimated by 15-20% due to the combination of scattering and refractive index layers [71]. However, as was shown in later studies [72], the local depth uncertainty is dependent upon the optical elements in the system and the refractive index of the material, which may vary amongst experiments. Notably, several mathematical models have been developed to simulate the laser beam distortions induced by refractive layers and light scattering effects in order to better estimate the depth and the resolution values [69,70,73]. However, these models tended to overestimate the depth when focusing deeper into the skin and, as noted by Tyali et al [72], all of these estimations are still “far from representing an accurate estimation” of the real depth value. Clearly, further work is required to evaluate local uncertainties in depth profiling in confocal Raman measurements on light-scattering tissues. Follow up investigation of this issue is currently underway in our laboratory. 

A key to practical use of Raman is whether it achieves both sufficient sensitivity and specificity for a particular application. The target sensitivity will depend on the concentrations of drug in the matrix of interest. For vaginal microbicides this would, for example, be maximum concentration in the delivery vehicle and much lower concentration in vaginal mucosal tissue harvested after *in vivo* dosing. In the evaluation of topical microbicides, confocal Raman spectroscopy could in principle perform several types of measurements, including: (1) drug release from test vehicles into tissue specimens; (2) drug permeation into and through tissue specimens; and (3) drug concentrations in specimens collected from pharmacokinetic studies. Clearly, drug concentrations in (3) would be lower than those encountered in *in vitro* experiments for (1) and (2), and this would necessitate a greater sensitivity. The three microbicide drugs studied here are believed to have different potencies in their anti-HIV activities, and their current loaded concentrations in clinical vaginal gels are consequently different. The standard concentration for Tenofovir in its clinical vaginal gel is 1% w/w. Its average concentration in human vaginal biopsy specimens has been about 0.01% w/w [36,58,62]. Prototype candidate clinical Dapivirine gels have been formulated with concentrations of 0.001, 0.005, 0.02, and 0.05% w/w [14,37]. A silicone matrix vaginal ring now in Phase 3 trials is loaded with 25 mg of Dapivirine, corresponding to a net concentration of about 0.31%w/w [39,40]. Dapivirine concentrations in human vaginal tissue biopsies for the gel trials were 0.0001- 0.0356%w/w. Mean Dapivirine concentration in human vaginal biopsy specimens in two phase I trials of intravaginal rings (IPM 001 and IPM 008) was in the range of 0.00003-0.00035%w/w. IQP-0528 has been formulated at a concentration of 0.25% in a gel [13], but no clinical trials of this gel have yet been conducted. More recently, a combination gel has been formulated and initially evaluated *in vitro*; this contains 2.5% Tenofovir and 1% IQP-0528 [12]. These various drug concentrations can be compared to the limits of detection (LOD) in the current study (Table 5). For Tenofovir, the LOD is about 25 - 100 fold lower than the concentration in the gel. The LOD for Dapivirine is about 8 - 17 fold lower than the concentration in the standard (0.05%) gel candidate. It is approximately 52 - 103 fold lower than the net concentration in the ring. The LOD for IQP-0528 is 6 - 25 fold lower than the current concentration formulated in the gel. Consequently, the current Raman configuration could be used for *in vitro* analyses of drug release from current microbicide vehicles (gels and rings) and for tissue permeation studies. 

The LOD for Tenofovir in current Raman set up here is about 4X higher than the concentration in the *in vivo* biopsy data for the 1% Tenofovir gel in clinical trials. However, we note that the LOD of Tenofovir in tissue is comparable to or lower than computational predictions (from a compartmental PK model) of Tenofovir concentrations in epithelium and upper stroma [18]. That is, to the extent that drug distributions in tissue follow diffusion theory, the time dependent concentrations of Tenofovir in upper layers may be much higher than average values determined from tissue biopsy punches. If so, then confocal Raman might be useful to measure Tenofovir concentrations in the upper mucosal layers of biopsy specimens. For Dapivirine, the LOD in tissue is from 6-fold lower to >17-fold higher than the concentrations measured in vaginal biopsies for the clinical trials. Moreover, the sensitivity of the Raman measurements of Tenofovir with the current instrument was sufficient to quantify concentration down to a depth of 200+ µm, and data of this kind could then be used in initial analyses of drug concentration distributions down into harvested vaginal mucosal tissues. 

Clearly, improvements are needed in the sensitivity of Raman-based measurements of microbicide drugs. The quantitative detection of compounds in complex, heterogeneous biological matrices, such as tissue, is generally confounded by the optical effects of out-of-focus light scattering and absorption. A key feature of the confocal approach is use of spatial filtering to reject the out-of-focus light from neighboring focal planes, by illuminating only one spot in a plane of focus through a pinhole. Implementation of confocal Raman imaging thus provides an improvement in axial resolution. In the current study, however, there was a trade-off between reasonable scanning times and achievable sensitivity of measurement. For Tenofovir, an integration time as low as 30 seconds was generally sufficient to measure high concentrations in tissue (> 0.1 %), while a scanning time as long as 5 minutes was required for detection near the LOD. Further improvements in the detection sensitivity would enable measurements of lower concentrations with reasonable scanning times, and would increase the depth down into tissue at which measurements could be made. 

In summary, we have presented an initial technical evaluation of the feasibility of applying Raman spectroscopy not only for qualitative identification, but also for quantitative analysis of local microbicide drug concentrations in fluids, clinical drug delivery gels and tissues. Results suggest that Raman offers promise for such applications. These can include analyses of biopsy specimens from *in vivo* PK studies, and measurements of drug transport *in vitro* from vehicles and fluids into excised tissue specimens. Implementation of confocal Raman spectroscopy improved the fidelity of z-axis measurements versus Raman as applied in a non-confocal configuration. Thus, the confocal Raman approach is the preferred modality in future studies of the kind here. Further, the Transwell configuration implemented here offers promise for measurement of concentrations and concentration profiles that can enable computation of traditional measures of drug release from vehicles and tissue permeability, and also computation of fundamental and incisive transport parameters – drug diffusion and partition coefficients in both target tissues and drug delivery vehicles. Such information can contribute to improved microbicide product design and evaluation.
